# A barley powdery mildew fungus non-autonomous retrotransposon encodes a peptide that supports penetration success on barley

**DOI:** 10.1093/jxb/ery174

**Published:** 2018-05-11

**Authors:** Mathias Nottensteiner, Bernd Zechmann, Christopher McCollum, Ralph Hückelhoven

**Affiliations:** 1 Chair of Phytopathology, TUM School of Life Sciences Weihenstephan, Technical University of Munich, Freising, Germany; 2 Center for Microscopy and Imaging, Baylor University, Waco, TX, USA

**Keywords:** *Blumeria graminis*, effector-triggered susceptibility, *Hordeum vulgare*, microtubule, retrotransposon, ROP GTPase, susceptibility factor, virulence

## Abstract

Pathogens overcome plant immunity by means of secreted effectors. Host effector targets often act in pathogen defense, but might also support fungal accommodation or nutrition. The barley ROP GTPase HvRACB is involved in accommodation of fungal haustoria of the powdery mildew fungus *Blumeria graminis* f.sp. *hordei* (*Bgh*) in barley epidermal cells. We found that HvRACB interacts with the ROP-interactive peptide 1 (ROPIP1) that is encoded on the active non-long terminal repeat retroelement Eg-R1 of *Bgh*. Overexpression of ROPIP1 in barley epidermal cells and host-induced post-transcriptional gene silencing (HIGS) of *ROPIP1* suggested that ROPIP1 is involved in virulence of *Bgh*. Bimolecular fluorescence complementation and co-localization supported that ROPIP1 can interact with activated HvRACB *in planta*. We show that ROPIP1 is expressed by *Bgh* on barley and translocated into the cytoplasm of infected barley cells. ROPIP1 is recruited to microtubules upon co-expression of MICROTUBULE ASSOCIATED ROP GTPase ACTIVATING PROTEIN (HvMAGAP1) and can destabilize cortical microtubules. The data suggest that *Bgh* ROPIP targets HvRACB and manipulates host cell microtubule organization for facilitated host cell entry. This points to a possible neo-functionalization of retroelement-derived transcripts for the evolution of a pathogen virulence effector.

## Teaser

A fungal retroelement encodes a peptide that translocates into the host cytoplasm, binds a host ROP GTPase, and supports fungal virulence. This suggests neo-functionalization of repetitive DNA in *Blumeria graminis*.

## Introduction

Considerable effort is invested in the understanding of plant immunity against infection by pathogens (Spoel and Dong, 2012) and the underlying genes such as resistance genes (*R*-genes) or quantitative trait loci (QTLs) that might be used in breeding for crops with improved resistance. In the general model, plant immunity towards invading pathogens is made up of two main layers, namely pattern-triggered immunity (Macho and Zipfel, 2014) and effector-triggered immunity (Spoel and Dong, 2012). Adapted pathogens evolved means to overcome host immunity, which is mainly attributed to secreted effector proteins that manipulate host cellular processes for the benefit of the pathogen. Plant hosts on the other hand evolved resistance proteins that directly or indirectly recognize the presence of a corresponding effector, or the action of effector proteins on their host targets, or on host decoy proteins that molecularly mimic host targets. Resistance protein signaling accelerates and increases defense responses typically resulting in the hypersensitive cell death response, thereby restricting further proliferation of biotrophic and hemibiotropic pathogens. The exerted mutual selection pressure drives co-evolution of host *R*-genes and pathogen effectors (Jones and Dangl, 2006).

The investigation of host factors that allow susceptibility against a pathogen is an alternative approach to searching for factors of host immunity. The products of susceptibility (*S*) genes might function in the regulation of plant defense responses or cell death. Alternatively, S-factors can be part of essential cellular processes from which the pathogen profits or that are co-opted by pathogens. The loss of function of *S*-gene products creates the chance for durable pathogen control due to the loss of a cellular function required for compatibility, given that possible pleiotropic effects are not detrimental for plant cultivation (for a review, see van Schie and Takken, 2014). A paradigm example for making use of the loss of *S*-gene functionality is the *MLO* gene, that represents a negative regulator of basal resistance against powdery mildews. Loss of MLO function is associated with powdery mildew resistance in diverse commercially important crop plant species (Kusch and Panstruga, 2017).

The ascomycete *Blumeria graminis* f.sp. *hordei* (*Bgh*) grows and reproduces on living host tissue where it causes barley powdery mildew. *Bgh* forms an appressorium and an infection peg for penetration of the host epidermis at 10–15 h after inoculation (hai). This penetrates and differentiates into a mature haustorium up to 48 hai. Haustoria stay separated from the host cell cytoplasm by the extrahaustorial matrix and a surrounding host membrane, the extrahaustorial membrane. In addition to expanding the surface for absorption of carbohydrates and amino acids (Voegele *et al.*, 2001), haustoria may serve for effector delivery into host cells. Penetration of the host cell is a prerequisite for further epicuticular development and asexual reproduction of *Bgh*.

The genomes of *Bgh* and of the close relative *Blumeria graminis* f.sp. *tritici* (*Bgt*) have been sequenced (Spanu *et al.*, 2010; Wicker *et al.*, 2013). Effector proteins of *B. graminis* are identified either via their avirulence (Avr) function if they are recognized by corresponding R-proteins or because of canonical characteristics of secreted effector proteins. *Bgh* encodes >500 candidate secreted effector proteins (CSEPs) (Pedersen *et al.*, 2012) identified by defined criteria for effector architecture. Some CSEPs are alternatively called BECs for *Blumeria* effector candidates, if they have been found to be expressed in *Bgh*-infected barley tissue (Bindschedler *et al.*, 2009; Pliego *et al.*, 2013). Recently, several CSEP proteins were shown to act as Avr factors in race-specific resistance of wheat and barley (Bourras *et al.*, 2015; Lu *et al.*, 2016; Praz *et al.*, 2017). *Bgh* also encodes 1350 paralogous copies of the second class of *Bgh* effector candidates, EKAs (effectors homologous to *Avrk1* and *Avra10*), which do not encode N-terminal signal peptides. The EKAs Avr_a10_ and Avr_k1_ are reported to be recognized by the corresponding barley R-proteins MLA10 and MLK1, respectively (Ridout *et al.*, 2006; Shen *et al.*, 2007; Nowara *et al.*, 2010). Avr_a10_ and Avr_k1_ evolved from 3'-truncated ORF1 proteins of *Bgh* long-interspersed element (LINE) retrotransposons (Amselem *et al.*, 2015). The ~120 Mb genome of *Bgh* and other powdery mildews is highly enlarged in comparison with the ascomycete mean, which was attributed to a high abundance of transposable elements (TEs). The genome of *Bgh* was estimated to be composed of ~65% TEs, and ~75% repetitive DNA content in total (Spanu *et al.*, 2010); >90% repetitive DNA content was estimated for *Bgt* (Wicker *et al.*, 2013). Both species show a substantial loss in gene number including genes for enzymes of primary and secondary metabolism. This might reflect their adaption to their obligate biotrophic lifestyle with a reduced gene set and some biological functions provided by the host.

The bulk of TE content in the *Bgh* genome are class I retrotransposons. Of these, non-long terminal repeat (LTR) retrotransposons are more abundant than the retrovirus-related LTR retrotransposons. Within non-LTR retrotransposons, autonomous LINEs are more abundant than non-autonomous short-interspersed elements (SINEs) that typically need LINE assistance for retrotransposition as they do not encode the required proteins. The SINE-classified non-LTRs Eg-R1 (Wei *et al.*, 1996) and Egh24 (Rasmussen *et al.*, 1993), for example, cover ~10% of the *Bgh* genome space (Spanu *et al.*, 2010).

The *Hordeum vulgare* (*Hv*) small monomeric Rho of plants (ROP) GTPase HvRACB has been shown to support *Bgh* haustorial ingrowth into barley epidermal cells when expressed as a constitutively activated (CA) mutant (Schultheiss *et al.*, 2003; Scheler *et al.*, 2016). Vice versa, RNAi-mediated silencing of *HvRACB* restricts haustorial invasion (Schultheiss *et al.*, 2002; Hoefle *et al.*, 2011; Scheler *et al.*, 2016). The activated GTP-bound HvRACB protein may thus support susceptibility. Two HvRACB-interacting barley proteins negatively regulate GTP-bound HvRACB. HvMAGAP1 is a microtubule- (MT) associated ROP-GTPase-activating protein (ROP-GAP) that apparently stimulates GTP hydrolysis depending on the catalytic arginine finger of its GAP domain (Hoefle *et al.*, 2011). Barley ROP-binding kinase1 (HvRBK1) is an active cytoplasmic receptor-like kinase, whose activity is stimulated by CA HvRACB *in vitro* and that directly binds to CA HvRACB *in planta* (Huesmann *et al.*, 2012). HvRBK1 in turn interacts with components of an E3 ubiquitin ligase complex and controls protein abundance of activated HvRACB (Reiner *et al.*, 2016).

Besides its role as an S-factor, HvRACB appears to function in polar cell growth processes (Hoefle *et al.*, 2011; Scheler *et al.*, 2016). Other plant ROP GTPases act in plant immunity (Kawano *et al.*, 2014). However, *HvRACB* apparently does not influence the ability of barley to express canonical PTI responses such as generation of reactive oxygen species (ROS) and phosphorylation of mitogen-activated protein kinases (Scheler *et al.*, 2016).

Here, we report on the HvRACB-interacting *Bgh* ROP-interactive peptide 1 (ROPIP1) that is encoded on the *Bgh* SINE-like retroposon Eg-R1. Our study suggests that ROPIP1 acts as a secreted intracellular virulence factor of *Bgh*.

## Materials and methods

### Plant growth and pathogen infection

Barley (*Hordeum vulgare* L.) cultivar ‘Golden Promise’ was grown at 18 °C, 60% relative humidity under a photoperiod of 16 h and a photon flux of 150 µmol s^–1^ m^–2^. *Blumeria graminis* (DC) Speer f.sp. *hordei* Em. Marchal, race A6 (Wiberg, 1974) was propagated on barley cultivar ‘Golden Promise’ under the same conditions. For protein extraction, 7-day-old barley plants were inoculated with >150 conidia mm^–2^ and left to grow until 10 days after inoculation (dai). The first leaves were inoculated with ~150 conidia mm^–2^ for reverse transcription–PCR (RT–PCR) and harvested at the indicated time points, or were inoculated with ~300 conidia mm^–2^ and left to grow until 3 dai for immunogold labeling and TEM. Transiently transformed detached 7-day-old primary leaves kept on 0.5% water–agar were inoculated with ~150 conidia mm^–2^ at 24 h after transformation (hat).

### Targeted Y2H

ROPIP1 was identified by DNA sequencing of positive prey clones from a yeast two-hybrid (Y2H) screen using HvRACB, CA HvRACB, and CA HvRAC1 as bait against a cDNA library prepared from *Bgh*-infected barley leaves, as in Hoefle *et al.* (2011). For targeted Y2H assays, yeast strain AH109 MATa was co-transformed with pGBKT7 bait plasmids and pGADT7 prey plasmids following the small-scale LiAc yeast transformation procedure (Clontech, Heidelberg, Germany).

ROPIP1-Nter was PCR-amplified from pGADT7-ROPIP1 using primers V42A_SmaI_F and R_V42A_Nter_BamHI (Supplementary Table S3 at *JXB* online), and *Sma*I/*Bam*HI cloned into pGADT7. ROPIP1-Cter was PCR-amplified from C-ROPIP1 using primers F_V42ACter_Sma and R_V42ACter_Bam, and *Sma*I/*Bam*HI cloned into pGADT7. Cloning of barley ROP proteins into the pGBKT7 vector is described in Schultheiss *et al.* (2008). Transformed cells were selected on SD medium lacking Leu and Trp (-L-W), resuspended in ultrapure water and spotted on SD-L-W and on interaction selective SD medium lacking Ade, His, Leu and Trp (-A-H-L-W). 3-Amino-1,2,4-triazole (3-AT) was optionally added in concentrations from 0.5 mM to 2.5 mM to the SD-A-H-L-W medium to increase selectivity.

### Transient transformation of barley leaf epidermal cells

Primary leaves of 7-day-old barley plants were cut and placed on solid 0.5% water–agar. Plasmids were coated to 1.0 µm gold particles (BioRad) and bombarded into barley epidermal cells using the PDS-1000/He (Bio-Rad) system as described earlier (Douchkov *et al.*, 2005; Eichmann *et al.*, 2010).

### Transient overexpression and HIGS

For transient overexpression, ROPIP1 and ROPIP1-Cter were PCR-amplified from cDNA using 5'-oligos V20A,V42ABamH1fwd and V42A,V20BBamH1kurz, respectively, and 3'-oligo V42A,V20Brev, A/T cloned into pGEM-T (Promega), and *Bam*HI/*Sal*I subcloned into the pUC18-based pGY1 plant expression vector (Trujillo *et al.*, 2006). A 5'-ATG start codon for ROPIP1 *in planta* expression was introduced into the ROPIP1 sequence by the 5'-oligo V20A,V42ABamH1fwd. Detached barley primary leaves were co-bombarded with 0.5 µg per shot of pGY1-GFP (green fluorescent protein) for the transformation control and 1.0 µg per shot of pGY1-ROPIP1 or pGY1-ROPIP1-Cter, or pGY1 empty vector. Microscopic evaluation of haustoria formation in GFP-fluorescing cells was at 48 hai. The relative penetration efficiencies were calculated by dividing the number of transformed cells with haustoria by the sum of susceptible plus resistant (attacked by *Bgh* but stopped) transformed cells of each combination. In each combination and repetition, at least 50 cell autonomous interactions were scored. The relative penetration rate was calculated by forming the quotient of the penetration efficiency of each sample divided by the penetration efficiency of the control. The variation of the control samples was calculated by dividing the penetration efficiency from each repetition by the arithmetic mean of all penetration efficiencies of the control samples. The arithmetic means calculated from the relative penetration efficiencies of the test samples were pairwise compared with the arithmetic means of the relative penetrations efficiencies of the control in a two-sided Student’s *t*-test.

For transient host-induced post-transcriptional gene silencing (HIGS), ROPIP1 was PCR-amplified from cDNA using the 5'-oligo V20A,V42ABamH1fwd and the 3'-oligo V42A,V20Brev, and blunt-ligated into the Gateway entry vector pIPKTA38. ROPIP1 was then recombined as an inverted repeat into the Gateway destination vector pIPKTA30N by a standard Gateway LR reaction (Douchkov *et al.*, 2005). The synthetic ROPIP1-RNAi-rescue (Eurofins MWG Operon) was designed by replacing the original codons by the most different but not rare barley codons (Supplementary Fig. 6C) as described by Nowara *et al.* (2010). The codon usage frequencies were obtained from the Codon Usage Database (http://www.kazusa.or.jp/codon/). ROPIP1-RNAi-rescue was *Bam*HI/*Sal*I subcloned from the delivered pEX-A2 plasmid into the pGY1 plant expression vector. GFP was cloned in-frame with ROPIP1-RNAi-rescue into the *Bam*HI cleavage site, resulting in pGY1-GFP-ROPIP1-RNAi-rescue. For the HIGS experiment, 1.0 µg per shot of pIPKTA30N-ROPIP1, or empty pIPKTA30N (control) plus either 1.0 µg per shot of pGY1-ROPIP1-RNAi-rescue or empty pGY1 and 0.5 µg per shot of pGY1-GFP each were bombarded into barley epidermal leaf cells. Assessment of fungal development on GFP-expressing cells took place at 48 hat, as described above for the overexpression experiment.

### Western blot

Total protein extracts from heavily *Bgh*-infected barley primary leaves or mock-treated control leaves were prepared using the Plant Total Protein Extraction kit (Sigma-Aldrich) following the manufacturer’s instructions. Around 200 mg of liquid N_2_-ground barley leaf powder was used for 250 µl of Protein Extraction Reagent Type 4. The protein concentration was determined by a Bradford assay. An aliquot of 50–100 µg of total protein per lane was separated by SDS–PAGE on hand-cast mini-gels (15% resolving gel, 4% stacking gel) using the Mini-PROTEAN Tetra Cell (Bio-Rad) in the Laemmli (Laemmli, 1970) buffer system; 200 V were applied for up to 45 min. Separated proteins were blotted onto 0.2 µm nitrocellulose membranes using a Fastblot B43 (Biometra) semi-dry blot system. A current of 5 mA cm^–2^ was applied for 25 min. Successful protein transfer was checked by Ponceau S staining. Nitrocellulose membranes were destained by two rounds of washing in 1× phosphate-buffered saline (PBS) for 10 min, before blocking in 5.0% non-fat dry milk in PBS for 1 h at room temperature. The blot was incubated with diluted primary antibodies (total barley protein extracts, 1:100; recombinant *Escherichia coli* crude lysates, 1:10 000) in blocking buffer overnight at 4 °C. After three rounds of washing in PBS-T each for 15 min, blots were incubated with anti-rabbit-horseradish peroxidase (Sigma-Aldrich) secondary antibodies diluted 1:80 000 in blocking buffer for 2 h at room temperature and washed again for three rounds. The SuperSignal West Femto Maximum Sensitivity Substrate (Thermo Scientific) was used as the ECL substrate. Chemiluminescence was documented with a Fusion-SL4 system operated with FusionCapt Advance Solo 4 (version 16.06) software. The custom antipeptide antibody α-ROPIP1 (Pineda Antibody Service, Berlin, Germany) was raised against the synthesized peptide NH_2_-IPSRLRDLYRLHF-COOH in rabbits in a 145 d custom-controlled immunization protocol and purified to ≥95% by affinity chromatography.

### Heterologous expression of recombinant ROPIP1

ROPIP1 was PCR amplified from plasmid using primers B8B,V21B_BamH1fwd and V42A,V20Bsalrev (Supplementary Table S3), and *Bam*HI/*Sal*I cloned into the pET28b(+) vector. The pET28b-ROPIP1-6His plasmid was further digested with *Nde*I/*Bam*HI to excise additional ATG start codons in the multiple cloning site (MCS). Sticky ends were blunted and the plasmid religated. The resulting pET28b-6His-ROPIP1-6His plasmid was transformed into chemically competent Rosetta (DE3) *E.* cells.

For crude -cell lysate preparation, 50 ml of LB Kan (50 µg ml^–1^ kanamycin) were inoculated with a 1:100-diluted overnight culture. Small-scale cultures were grown until they reached an OD_600_ of 0.8–1.0. Non-induced aliquots were taken. Recombinant protein expression was induced by addition of isopropyl-β-d-galactopyranoside (IPTG) to a final concentration of 1 mM. Induced and parallel non-induced cultures were grown at 37 °C for an additional 1–3 h. Crude cell lysates were prepared by resuspending bacterial pellets in 100 µl of Lysis Buffer (50 mM NaH_2_PO_4_-H_2_O, 300 mM NaCl, 10% glycerol, 1% Triton X-100, 1 mg ml^–1^ lysozyme, pH 8.0) per 1 ml of culture volume and incubation on ice for 30 min. Three rounds of ultrasonic bath incubation for 10 s followed, placing the lysates on ice in between each round. Viscosity of lysates was reduced by addition of 50 U of Benzonase (Merck Millipore) per 1 ml of culture volume and a further incubation on ice for 15 min. Up to 10 µl of heat-denatured crude lysate were loaded per lane onto SDS–polyacrylamide gels. Non-induced control samples and IPTG-induced samples were run as duplicates on the same gels followed by western blotting. Afterwards, one half of the nitrocellulose membrane was incubated with α-ROPIP1 as primary antibody and the duplicate half was incubated with anti-His-Hrp (Carl Roth).

RecROPIP1 was purified with the Protino Ni-TED 2000 packed columns kit (Macherey Nagel) following the batch gravity-flow purification protocol under native conditions (User Manual, version Rev.04, protocol 5.5).

### Immunocytohistochemical detection of α-ROPIP1

Sample preparation for TEM and immunogold labeling was performed according to a modified version described previously (Redkar *et al.*, 2015). Briefly, samples were fixed with 2.5% (w/v) paraformaldehyde and 0.5% (v/v) glutaraldehyde in 0.06 M Sørensen phosphate buffer, then rinsed in buffer, dehydrated in acetone, and embedded in LR-White resin (London Resin). Immunogold labeling of α-ROPIP1 was performed on ultrathin sections with an automated immunogold labeling system (Leica EM IGL, Leica Microsystems). The sections were blocked for 20 min with 2% (w/v) BSA (Sigma-Aldrich) in PBS, pH 7.2, and then treated with the primary antibody α-ROPIP1 against ROPIP1 for 90 min diluted 1:100 in PBS containing 1% (w/v) BSA. After sections were washed twice for 5 min with PBS containing 1% (w/v) BSA, they were treated with a 10 nm gold-conjugated secondary antibody (goat anti-rabbit IgG, British BioCell International) diluted 1:100 in PBS containing 1% (w/v) BSA for 90 min. After a short wash in PBS (3 × 5 min), labeled grids were post-stained with 2% uranyl acetate aqueous solution for 15 s and then investigated with a Philips CM10 transmission electron microscope. The ideal dilutions and incubation times of the primary and secondary antibodies were determined in preliminary studies by evaluating the labeling density after a series of labeling experiments. The final dilutions used in this study showed a minimum background labeling outside the sample with a maximum specific labeling in the sample. Various negative controls were performed to confirm the specificity of the immunocytohistochemical approach. Gold particles were absent on sections when (i) no primary antibody; (ii) a non-specific secondary antibody (goat anti-mouse IgG); and (iii) pre-immune serum instead of the primary antibody was used.

### Live cell imaging

Transiently transformed barley epidermal leaf cells expressing fluorophore fusion proteins were imaged with a Leica TCS SP5 confocal laser scanning microscope using standard wavelengths for excitation and emission. Barley epidermal cells were scanned as *z*-stacks in 2 µm increments in sequential scan mode. Maximum projections were exported from the Leica LAS AF software (version 2.5.1) in jpeg or tiff format.

### Quantification of GFP–ROPIP1 MT localization and destruction

GFP was cloned in-frame with ROPIP1 into the 5'-*Bam*HI restriction site of pGY1-ROPIP1 to produce pGY1-GFP-ROPIP1. The cloning of pGY1-RFP-HvMAGAP1 and variants is described in Hoefle *et al.* (2011). Barley epidermal cells were transiently transformed with 0.5 µg per shot of pGY1-GFP or 0.75 µg per shot of pGY1-GFP-ROPIP1 plus 1.0 µg per shot of pGY1-RFP-HvMAGAP1 or 1.0 µg per shot of pGY1-RFP-HvMAGAP1-Cter and imaged as whole-cell scans with 2 µm increments at 12–24 hat. For quantification of MT localization of GFP–ROPIP1, cells were categorized into GFP signal present at MTs or absent from MTs. The numbers of categorized cells were compared between cells co-expressing red fluorescent protein (RFP)–HvMAGAP1 or RFP–HvMAGAP1-Cter together with GFP–ROPIP1 in a χ^2^ test with df=1. For quantification of the MT network organization, maximum projections were categorized into intact, disordered, or fragmented MTs. The distribution of the absolute cell numbers per category was compared between cells co-expressing GFP or GFP–ROPIP1 along with RFP–HvMAGAP1 in a χ^2^ test with df=2.

### Bimolecular fluorescence complementation

ROPIP1 was PCR-amplified from plasmid using 5'-oligo V20A,V42ABamH1fwd and 3'-oligo V42A,V20Bsalrev, and *Bam*HI/*Sal*I cloned into the MCS of pUC-SPYNE (Walter *et al.*, 2004) which translated into ROPIP–YFP^N^. The cloning of pUC-SPYCE-CA HvRACB and pUC-SPYCE-DN HvRACB, both translating into an N-terminal fusion of YFP^C^ to CA/DN (dominant negative) HvRACB, is described in Schultheiss *et al.* (2008).

Barley leaf epidermal cells (7 d old) were transiently co-transformed with 0.75 µg per shot of pUC-SPYNE-ROPIP1 plus 0.75 µg per shot of pUC-SPYCE-CA HvRACB and pUC-SPYCE-DN HvRACB, 0.5 µg per shot of pGY1-CFP, and 1.0 µg per shot of pGY1-RFP-HvMAGAP1-R185G. Transformed cells were identified by cyan fluorescent protein (CFP) fluorescence and imaged by confocal laser scanning microscopy at 36 hat. Each fluorophore was excited and detected in an individual scan by sequentially scanning between frames. All hardware and software settings were kept identical for all cells and repetitions.

The bimolecular fluorescence complementation (BiFC) signal was analyzed in a quantitative manner using maximum projections of transformed cells and the Leica LAS AF (version 2.5.1.6757) ‘Quantify’ tool. The first region of interest (ROI 1) was put at the cell periphery of the transformed cell. The second, copy-pasted, ROI 2, was placed into the surrounding background close to the cell. The mean values of fluorescence intensity of the ROIs (mean fluorescence intensity, MFI) of the yellow fluorescent protein (YFP) and the CFP detector were read out from the quantification reports. The background fluorescence MFI (ROI 2) was subtracted from ROI 1. The corrected MFI of the YFP detector was divided by the corrected MFI of the CFP detector. The obtained YFP/CFP MFI ratios of YFP^C^–CA HvRACB- and YFP^C^–DN HvRACB-co-expressing cells were compared in a two-sided Student’s *t*-test. The corrected CFP MFIs were also compared in a two-sided Student’s *t*-test and did not differ.

### 5'-RACE-PCR

The Dynabeads mRNA Direct Kit (Thermo Scientific) was used according to the manufacturer’s instruction for isolation of poly(A) RNA from *Bgh*-infected barley primary leaves. After DNase I digestion, the isolation process was repeated. A 0.5–1.0 µg aliquot of poly(A) RNA was reverse-transcribed into first-strand cDNA following the instructions of the 5'/3' RACE kit, 2nd Generation, version 12 (Roche) and using the oligo TW42A_R as the cDNA synthesis primer (Supplementary Table S3). The resulting dA-tailed cDNA was used as template for PCR amplification using Phusion High-Fidelity DNA Polymerase (Thermo Scientific) and V42A-SP2 as the gene-specific primer. V42A-SP3 was used as the nested gene-specific primer in a second PCR run. PCR products were gel purified, A-tailed, cloned into pGEM-T (Promega), and sequenced.

### Semi-quantitative RT–PCR

*Bgh*-inoculated and mock-treated barley primary leaves (7 d old) were cut and immediately frozen in liquid N_2_. Total RNA was prepared (Chomczynski and Sacchi, 1987), precipitated by NaAc/ethanol to achieve greater purity, and digested with DNase I (Thermo Scientific). First-strand cDNA was synthesized with RevertAid Reverse Transcriptase (Thermo Scientific) using oligo(dT)_15_ primer (Promega). The barley *Ubiquitin Conjugating Enzyme 2* (*HvUBC2*; AY220735.1) gene was amplified using the oligo pair HvUBC2_fwd and HvUBC2_rev. The barley *Basic PR-1-Type Pathogenesis Related Protein* (*HvPR1b*; X74940.1) gene was amplified using the oligo pair T-PR1b/5'-2 and T-PR1b/3'-2. The *Bgh Tub2 Gene For Beta Tubulin* (*Bgh tub2*; AJ313149) gene was amplified using the oligo pair *Bgh*_beta-tub_F and *Bgh*_beta-tub_R. *Bgh ROPIP1* transcript was amplified using the oligo pair V42fwd and V42rev.

## Results

### ROPIP1 is encoded by the retrotransposable element Eg-R1 of *Bgh*

We performed Y2H screens using the barley ROPs HvRACB (GenBank accession number: AJ344223), CA HvRACB, and CA HvRAC1 (GenBank accession number: AJ518933) as baits against a cDNA library prepared from *Bgh*-infected barley leaves. Besides the barley proteins, HvMAGAP1 and HvRBK1 (Hoefle *et al.*, 2011; Huesmann *et al.*, 2012), a *Bgh*-derived cDNA was repeatedly isolated (twice with HvRACB, four times with CA HvRACB, and twice with CA HvRAC1). Sequencing of the respective plasmids isolated from yeast retrieved a polyadenylated transcript and fragments of the same transcript that aligned to its 5' region. Initial BLAST searches against the NCBI nucleotide database identified the transcript as the non-LTR retroelement Eg-R1 (Wei *et al.*, 1996) (GenBank accession number: X86077.1) of *Bgh*. The Eg-R1 5' sequence as obtained from the fragments in-frame with the activation domain of the prey vector would give rise to a 74 amino acids peptide (Supplementary Fig. S1) which interacted with HvRACB, CA HvRACB, and CA HvRAC1 in the bait vectors. Later on, we named this peptide ROP-INTERACTIVE PEPTIDE 1 (ROPIP1) of *Bgh*.

A BLAST search of the Eg-R1 nucleotide sequence against the assembled *Bgh* reference genome [BGH DH14 Genome v3b (contigs); https://genome.jgi.doe.gov/Blugr1/Blugr1.home.html] of race DH14 suggested >3000 genomic insertions of the Eg-R1 element and similar numbers in other *Bgh* races (Hacquard *et al.*, 2013). This number is probably underestimated as, for example, only half of the genome of *Bgh* race A6 was assembled due to the high repeat content (Hacquard *et al.*, 2013). We randomly selected 53 full-length Eg-R1 genomic insertions for inspection of the direct genomic environment. Interestingly, eight of the 53 insertions showed 5'-elongated ORFs including the 74 amino acids that had been isolated in the Y2H screening in-frame with predicted signal peptides for secretion (SignalP 3.0 Server). This prediction dropped to two predicted signal peptides in 53 chimeric ORFs with the stringent settings of SignalP4.1 (Supplementary Table S1). This would extrapolate to many of such genomic sequences given at least 3000 genomic insertions. 5'-RACE-PCR further confirmed (Supplementary Table S2) the recently published Eg-R1 consensus sequence (Eg-R1_cons) (Amselem *et al.*, 2015). BLAST searches of the ROPIP1 or the Eg-R1 nucleotide sequence against the NCBI nucleotide collection exclusively produced hits matching to the species *B. graminis*, possibly hinting at a specificity of the Eg-R1 element for powdery mildews of *Poaceae*. A highly similar retroelement, Bgt_RSX_Lie, was identified in the genome of the close *Bgh* relative *Bgt* of wheat (Parlange *et al.*, 2011).

Eg-R1 was originally described as a repetitive element that shares some features with SINEs but which is also distinct from classical SINEs (Wei *et al.*, 1996). SINEs typically share sequence similarities with tRNAs, 7SL RNA, or 5S rRNA from which they may derive (Kramerov and Vassetzky, 2011). All these are transcribed by RNA polymerase III. As reported by Wei and colleagues (1996), Eg-R1 lacks A-box and B-box RNA polymerase III transcription initiation sites within its 5' region. Furthermore, internal poly(T) stretches would act as RNA polymerase III termination signals such that a RNA polymerase III transcript would be truncated, which renders transcription by RNA polymerase III very unlikely. Genomic insertions of Eg-R1 lacked genomic poly(A)-coding stretches at their 3' ends but comprised a 5'-AAUAAA-3' polyadenylation signal, which is obviously functional since Eg-R1 is expressed as polyadenylated RNA (Wei *et al.*, 1996; Supplementary Fig. S1; see Supplementary Fig. S2 for Eg-R1 architecture). This supports protein-coding gene-like transcription of Eg-R1 by RNA polymerase II. The ROPIP1 nucleotide sequence was amplifiable from cDNA prepared from total RNA extracts as well as from poly(A) mRNA preparations of *Bgh*-inoculated barley leaves but not from the non-inoculated control (Supplementary Fig. S3). Wei and colleagues (1996) detected Eg-R1 on a northern blot of poly(A) RNA (Wei *et al.*, 1996). Expression of ROPIP1 and Eg-R1 was further supported by BLAST searches against ESTs of *Bgh* (BGH DH14 All ESTs database) of race DH14 (https://genome.jgi.doe.gov/Blugr1/Blugr1.home.html) and RNAseq data of *Bgh* race A6 grown on the immunocompromised *Arabidopsis thaliana* (Hacquard *et al.*, 2013). Genomic insertions of Eg-R1 were found located in the close spatial vicinity of CSEPs, where Eg-R1 was suggested to contribute to unequal crossing over events (Pedersen *et al.*, 2012). This might be supported by our finding of truncated Eg-R1 genomic insertions not being reflected by preferential insertion of an Eg-R1 partial sequence, which could have arisen from, for example, incomplete insertion of the element (Supplementary Fig. S4A, B). Eg-R1 is deposited at Repbase (Repbase Report 2011, Volume 11, Issue 9; Jurka *et al.*, 2005) as one member of a family of eight *B. graminis* non-LTR retrotransposons (BG_Non-LTRs), which were found to be conserved in their 5' region (Supplementary Fig. S4C, D). In summary, the ROPIP1 sequence was found encoded on Eg-R1, which is probably a member of a class of as yet not well characterized, non-autonomous, RNA polymerase II-transcribed retroelements.

### ROPIP1 interacts with barley susceptibility factor HvRACB in yeast

**Fig. 1.**
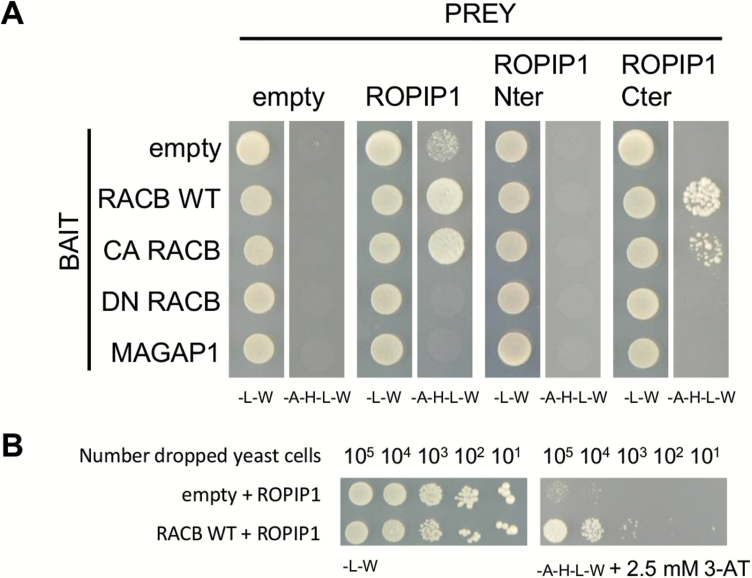
*Bgh* ROPIP1 and ROPIP1-Cter interacted with barley HvRACB and CA HvRACB in yeast. (A) ROPIP1 of *Bgh* was tested as prey in targeted Y2H assays for interaction with the barley small GTPase HvRACB in three different variants: WT, wild-type protein; CA, constitutively activated mutant (HvRACB G15V); DN, dominant negative mutant (HvRACB T20N) and with the HvRACB-interacting protein HvMAGAP1. The ROPIP1 sequence was additionally split into its small inherent C-terminal ORF (ROPIP1-Cter) which was sufficient for protein interaction with WT HvRACB and CA HvRACB and the remaining N-terminal part (ROPIP1-Nter) which did not interact with the baits. A total of 10^5^ cells of each combination were dropped in parallel on SD-Leu,-Trp (-L-W) as transformation control and on SD-Ade,-His,-Leu,-Trp (-A-H-L-W) selection medium. (B) Serial dilution of 10^5^–10 yeast cells transformed with pGADT7-ROPIP1 as prey vector and pGBKT7-HvRACB WT as bait vector or pGBKT7-empty as empty vector control. Left panel: transformation control medium (SD-L-W). Right panel: selection medium (SD-A-H-L-W) supplemented with 2.5 mM 3-AT to increase selectivity. (This figure is available in colour at *JXB* online.)

We next verified the ROPIP1–HvRACB protein interaction in yeast by independent targeted Y2H assays. Besides the wild-type and CA HvRACB, the dominant negative mutant DN HvRACB and HvMAGAP1 (GenBank accession number: AK371854) were additionally included as bait proteins. Yeast colony growth of the prey–bait combinations ROPIP1–HvRACB and ROPIP1–CA HvRACB exceeded all other combinations on interaction-selective media (Fig. 1A; Supplementary Fig. S5A). Weak background growth of the ROPIP1 prey was abolished when plating yeast on 2.5 mM 3-AT (Fig. 1B). No colony growth was observable when ROPIP1 was combined with either DN HvRACB or HvMAGAP1. ROPIP1 also did not interact (for CA HvRACD, CA HvROP6, and CA HvRAC3) or weakly interacted with other barley ROP baits (HvRAC1 and CA HvRAC1) (Supplementary Fig. S5A). There is no obvious ATG start at the very 5' end of the Eg-R1 nucleotide sequence we found in the Y2H screening. However, there is an ORF in the same reading frame of the ROPIP1 sequence, which translates into a shorter peptide of 44 amino acids and which we refer to as ROPIP1-Cter (Supplementary Fig. S1). Interestingly, the ATG start and TGA stop codons of ROPIP1-Cter are present in the majority of Eg-R1 full-length genomic insertions (see, for example, Supplementary Table S2 for the Eg-R1 consensus sequence) but not conserved in the other Bg-non-LTRs. In order to delimit the HvRACB-interacting part, ROPIP1 was split into ROPIP1-Cter and the remaining N-terminus (ROPIP1-Nter). The fragments were tested against the same baits as ROPIP1 in targeted Y2H assays. ROPIP1-Cter in the prey vector did not show any background growth. ROPIP1-Cter interacted in yeast with CA HvRACB and HvRACB, but not with DN HvRACB, which was similar to ROPIP1 as prey. However, colonies grew less dense when compared with ROPIP1 (Fig. 1A), and no interaction was observed for ROPIP1-Cter with HvRAC1 or CA HvRAC1 (Supplementary Fig. S5B). ROPIP1-Nter was not sufficient for interaction with any of the baits. Together, these findings suggest that binding of ROPIP1 to HvRACB is largely mediated by ROPIP1-Cter. Secondary structure prediction for ROPIP1 proposed folding in α-helices and β-sheet structures (Supplementary Fig. S4E, F).

### ROPIP1 enhances virulence of *Bgh*

**Fig. 2.**
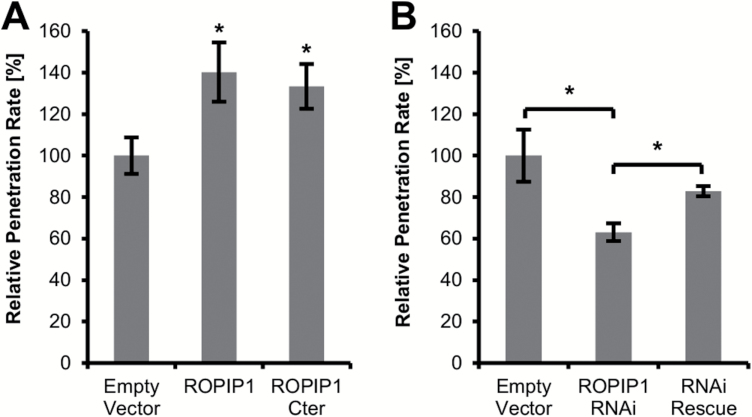
ROPIP1 modulated susceptibility of barley epidermal cells towards *Bgh*. (A) Transient overexpression of ROPIP1 and ROPIP1-Cter in barley epidermal cells significantly increased the relative penetration rate of *Bgh* in comparison with the control. (B) Host-induced gene silencing (HIGS) of native *ROPIP1* by transient expression of ROPIP1 as dsRNA (ROPIP1-RNAi) in barley epidermal cells significantly decreased the relative penetration rate of *Bgh*. Co-expression of a ROPIP1-RNAi-rescue construct (RNAi rescue) significantly complemented HIGS of the native *ROPIP1* transcript. Bars represent the mean values of six independent experiments in (A) and four independent experiments in (B). Error bars are ±SE. *P≤0.05 (Student’s *t*-test).

As ROPIP1 interacted with the S-factor HvRACB, we checked whether ROPIP1 can affect the susceptibility of barley against *Bgh*. Therefore, we transiently expressed ROPIP1 in barley epidermal cells by microprojectile bombardment prior to inoculation with *Bgh* conidial spores at 24 hat and microscopic analysis of fungal development at 48 hai. To express the full ROPIP1 sequence including the ROPIP1-Nter and ROPIP1-Cter *in planta*, we equipped the sequence with an additional ATG start codon at its very 5' end (Suplementary Tables S1, S2). Transformed cells were identified by co-bombarded GFP. Overexpression of ROPIP1 led to a significant increase (*P*≤0.05, Student’s *t*-test) in susceptibility to fungal penetration of transformed barley leaf epidermal cells. This was evident from an enhanced frequency of attacked cells with fungal haustoria. Hence, ectopic expression of ROPIP1 promoted virulence of *Bgh* (Fig. 2A). The relative penetration rate increased thereby by ~40%. Ectopic overexpression of ROPIP1-Cter in barley epidermal cells had an effect comparable with albeit somewhat weaker than that of ROPIP1. This added to the view of ROPIP1-Cter being the part of ROPIP1 that promotes virulence of *Bgh*.

Ectopic expression of double-stranded RNAi constructs in barley epidermal cells proved to be a valuable tool for silencing *Bgh* transcripts in a process called HIGS (Nowara *et al.*, 2010; Zhang *et al.*, 2012; Pliego *et al.*, 2013; Ahmed *et al.*, 2015). ROPIP1 was hence cloned as an inverted repeat into the plant RNAi vector pIPKTA30N (Douchkov *et al.*, 2005). Off-target prediction using the SI-FI software (Nowara *et al.*, 2010) did not reveal further targets in *Bgh* or in barley. For the HIGS experiment, the transformed leaves were inoculated at 24 hat with *Bgh* conidia followed by microscopic analysis of fungal development at 48 hai. HIGS of ROPIP1 significantly (*P*≤0.05, Student’s *t*-test) reduced the relative penetration rate of *Bgh* on transformed cells by 38% (Fig. 2B). We included a synthetic ROPIP1 RNAi-insensitive rescue construct (Supplementary Fig. S6C) in the experiment to ensure that the observed drop in virulence of *Bgh* was due to post-transcriptional silencing of ROPIP1. The functionalities of the ROPIP1-RNAi and ROPIP1-RNAi-rescue constructs were tested in advance by transient co-expression experiments and silencing of GFP–ROPIP fusion constructs (Supplementary Fig. S6A). Accordingly, ROPIP1-RNAi-rescue partially but significantly (*P*≤0.05, Student’s *t*-test) rescued the ROPIP1-RNAi-mediated decrease in fungal penetration success (Fig. 2B).

### ROPIP1 protein is detectable in *Bgh*-infected barley leaf protein extracts

**Fig. 3.**
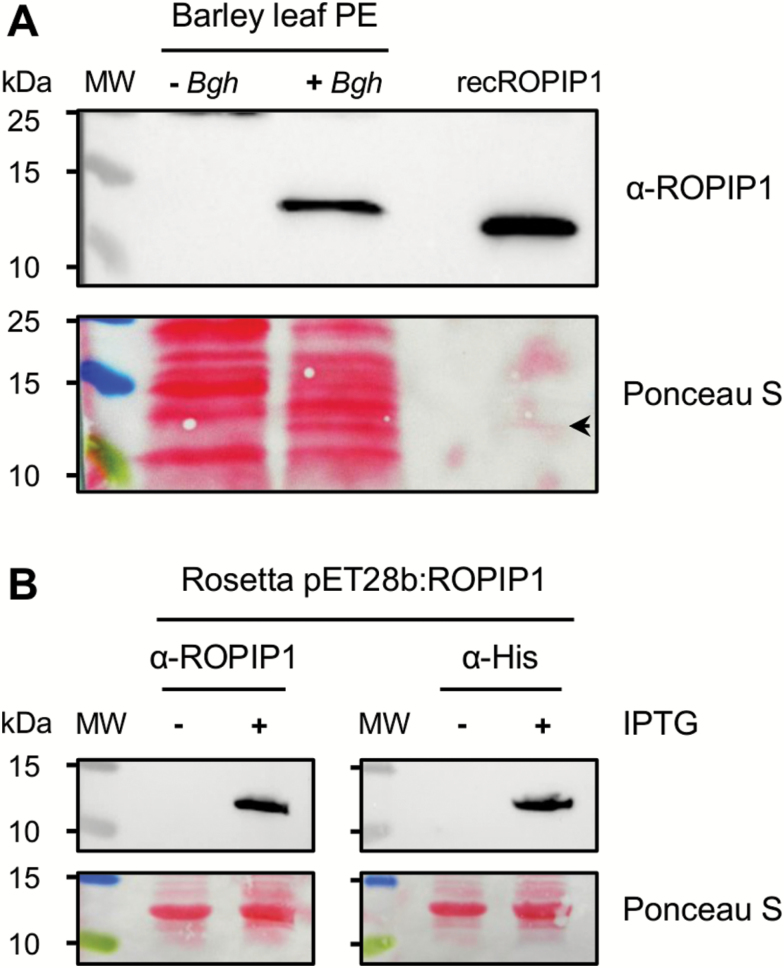
Western blot of barley leaf protein extracts using α-ROPIP1 antibody. (A) Affinity-purified anti-peptide antibody α-ROPIP1 was used as the primary antibody in western blots of total protein extracts prepared from barley leaves inoculated (+*Bgh*) or non-inoculated (–*Bgh*) with *Bgh*. His-tag purified recombinant ROPIP1 (recROPIP1) was run as a positive control on the same gel. RecROPIP1 and a protein exclusive to the +*Bgh* sample were labeled by α-ROPIP1. Several repetitions confirmed the signal in the +*Bgh* lane. (B) Controls for α-ROPIP1 specificity. *Escherichia coli* Rosetta cells were transformed with the IPTG-inducible vector pET28b:ROPIP1. Crude cell lysates were prepared from small-scale cell cultures with (+) or without (–) IPTG induction. Recombinant His-tagged ROPIP1 was detected by α-ROPIP1 in the IPTG-induced sample (+) but not in the non-induced control (–). The use of α-His antibody in aliquots of the same samples validated the identity of the signal. The experiment was repeated twice with identical results. Ponceau S: loading and protein transfer control. The arrowhead points to a faint band in the recROPIP1 lane in (A). MW, molecular weight protein ladder; PE, protein extract. (This figure is available in colour at *JXB* online.)

We next investigated whether a native ROPIP1 protein is detectable. A custom rabbit polyclonal antibody, α-ROPIP1, was raised against a synthesized epitope peptide derived from ROPIP1-Cter. The monospecific IgG fraction was purified to ≥95% by affinity chromatography using the epitope peptide as antigen. Total protein extracts were prepared from heavily *Bgh*-infected and non-inoculated barley primary leaves. A unique band in the protein extract of the *Bgh*-inoculated sample was repeatedly observable in a series of western blots (Fig. 3A). The band was never seen in the protein extract prepared from non-inoculated samples. Recombinant, *E. coli*-expressed His-tagged ROPIP1 (recROPIP1) was run as a positive control on the same gel and was detected by α-ROPIP1 (Fig. 3A). Further, α-ROPIP1 specifically detected recROPIP1 in crude cell lysates of *E. coli* cell cultures following induction of recombinant protein expression with isopropyl-β-d-1-thiogalactopyranoside (IPTG). The identity of the signal was confirmed by, first, the absence of the band in the non-induced control, and secondly by probing aliquots of the same crude cell lysates with an independent α-His antibody, which resulted in an identical signal pattern (Fig. 3B).

### TEM localizes ROPIP1 in *Bgh* structures and in the host cell cytoplasm.

Next, we analyzed the localization of the protein labeled by α-ROPIP1 *in situ*. We used immunogold labeling and TEM. Ultrathin cuts of heavily *Bgh*-infected (3 dai) barley primary leaves were incubated with α-ROPIP1 or an unspecific antibody as primary antibodies. Primary antibodies were detected by anti-rabbit secondary antibodies conjugated to 10 nm gold particles.

**Fig. 4.**
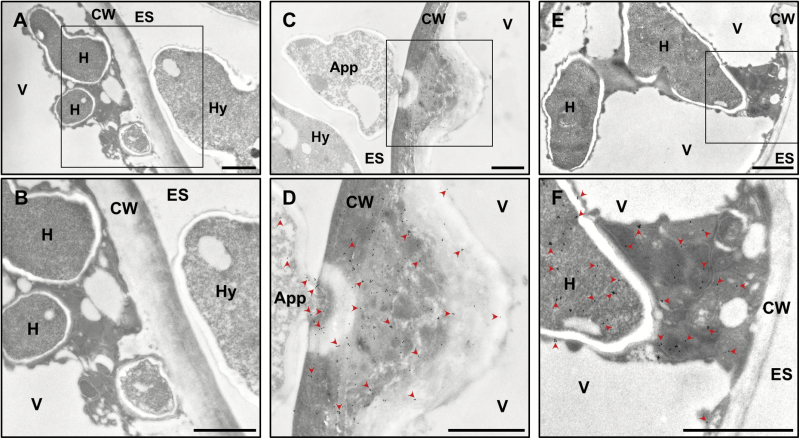
Immunogold labeling of α-ROPIP1 in *Bgh*-challenged barley leaves. Transmission electron micrographs of ultrathin sections of *Bgh*-infected barley epidermal cells 3 dai showing gold particles bound to α-ROPIP1. (A, B) Negative control of infected cells treated with a non-specific antibody. Gold particles were absent in the susceptible barley epidermal cell containing intracellular fungal haustorial protrusions (H) and the extracellular *Bgh* hypha (Hy). (C, D) Gold particles bound to α-ROPIP1 were observed in hyphae, inside a *Bgh* appressorium (App), the barley epidermal cell wall (CW), and papilla, but were absent from the extracellular space (ES) and the host cell vacuole (V). (E, F) Gold particles were found in the lumen of finger-like *Bgh* haustorial protrusions inside barley epidermal cells as well as the host cell cytoplasm, but were almost absent from the host cell vacuole (V), the CW, and the ES. Arrowheads in (D) and (F) point to selected gold particles. Scale bars are 1 µm.

Fungal intra- and extracellular structures, the extracellular space, the cell wall, and the barley epidermal cell interior were almost free from gold particles in the unspecific antibody control (Fig. 4A, and detail in B). In contrast, gold particles labeled fungal and host cell structures when using of α-ROPIP1 as primary antibody. In a barley epidermal cell, showing a host cell wall apposition (CWA; also called a papilla), gold particles were found in the epicuticular fungal hyphae, the appressorium, inside the host cell wall, and the host CWA (Fig. 4C, and detail in D). Gold particles appeared to spread from the tip of the appressorium but were almost absent from the extracellular space and the host cell vacuole. Hence, α-ROPIP1 obviously targeted a secreted fungal protein. In a penetrated barley epidermal cell, where *Bgh* established a fungal haustorium, gold particles were located in the fungal haustorium as well as in the host cytoplasm (Fig. 4E, and detail in F) but not in the host vacuole, showing that epitopes were not displaced during sample preparation. Therefore, the α-ROPIP1-labeled protein apparently was able to translocate from the fungus into the cytoplasm of barley epidermal host cells. Almost no gold particles were detectable in mesophyll cells of *Bgh*-infected barley leaves. Very few gold particles were occasionally observed in plastids (Supplementary Fig. S7).

In sum, immunogold labeling with α-ROPIP1 detected a secreted *Bgh* protein that translocated from the fungus into barley epidermal cells, where it could interact with HvRACB.

### HvRACB binding HvMAGAP1 recruits ROPIP1 to microtubules

**Fig. 5.**
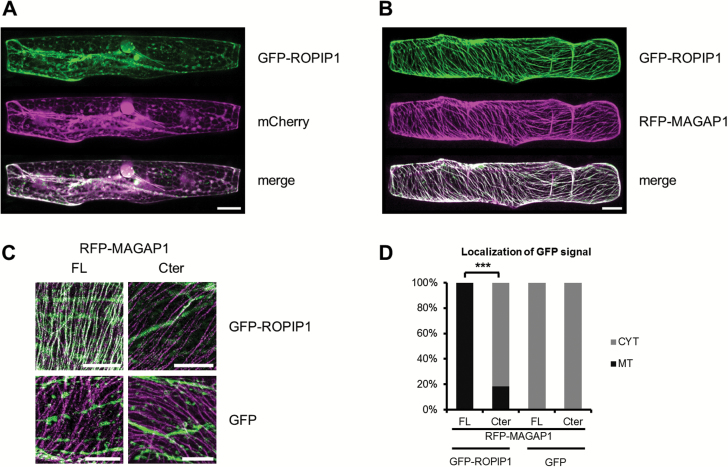
Recruitment of GFP–ROPIP1 to cortical microtubules (MTs) by RFP–HvMAGAP1. Barley leaf epidermal cells were transiently transformed by particle bombardment and imaged with confocal laser scanning microscopy as sequential whole-cell scans in 2 µm increments at 12–24 hat. (A) Whole-cell projection showing cytoplasmic and unspecific subcellular localization of GFP–ROPIP1. Co-localization with cytoplasmic and nucleoplasmic mCherry fluorescence is indicated by white pixels in the merge picture. The observation was consistently repeatable in more than three experiments. (B) Recruitment of GFP–ROPIP1 to cortical MTs upon co-expression of MT-associated RFP–HvMAGAP1. White pixels in the merge picture indicate co-localization. A maximum projection of 20 optical sections in 2 µm increments is shown. The observation was consistently repeatable in more than three experiments. (C) Visualization of co-expressed fusion protein combinations used for quantitative analysis. C-ter, truncation of HvMAGAP1 to the MT-associated C-terminus (HvMAGAP1-Cter); FL, full-length HvMAGAP1. Ten optical sections of the upper cell cortex were merged for the pictures. (D) Quantification of the combinations shown in (C). Bars are frequencies of cells with GFP fluorescence being located at MTs or in the cytoplasm only (CYT) derived from three independent experiments. The respective absolute numbers of the categories were compared in a χ^2^ test. RFP–HvMAGAP1-Cter highly significantly reduced MT association of GFP–ROPIP1 (***P≤0.001, *n*=61, 60, 53, and 57 cells from left to right). Scale bars in (A), (B), and (C) are 20 µm.

With ROPIP1 being a potential intracellular effector of *Bgh*, we progreesed to live cell imaging of GFP-tagged ROPIP1 by confocal laser scanning microscopy. Transient expression of GFP–ROPIP1 in barley epidermal cells did not show a distinct subcellular localization of ROPIP1. GFP–ROPIP1 labeled the cytoplasm and the nucleus (Fig. 5A). This was in line with the ROPIP1 sequence not showing any predictable cellular localization signatures or protein domains. As HvRACB-interacting proteins associate with MTs or function in regulation of MT network stability, we expressed GFP–ROPIP1 together with the putative HvRACB regulator HvMAGAP1 that has a unique localization at MTs (Hoefle *et al.*, 2011). Although ROPIP1 did not interact with HvMAGAP1 in yeast (Fig. 1), GFP–ROPIP1 was recruited to MTs under co-expression of red fluorescing RFP–HvMAGAP1 (Fig. 5B). The C-terminus of HvMAGAP1 (HvMAGAP1-Cter) mediates MT association of HvMAGAP1 but does not interact with HvRACB because it lacks the ROP-interacting CRIB motif and the GAP domains (Hoefle *et al.*, 2011). Quantification of subcellular fluorescence of GFP–ROPIP1 at 12–24 hat revealed that full-length RFP–HvMAGAP1 recruited GFP–ROPIP1 to MTs whereas RFP–HvMAGAP1-Cter hardly co-localized with GFP–ROPIP1 at MTs (*P*≤0.001, χ^2^, Fig. 5C, D). Instead, GFP–ROPIP1 labeled the cytoplasm, as did soluble GFP upon co-expression of RFP–HvMAGAP1 or RFP–HvMAGAP1-Cter (Fig. 5C, D). Hence, GFP–ROPIP1 localization at cortical MTs depended on RFP–HvMAGAP1 with its corresponding HvRACB-binding domains.

### ROPIP1 and CA HvRACB interact *in planta* and can co-localize with HvMAGAP1

**Fig. 6.**
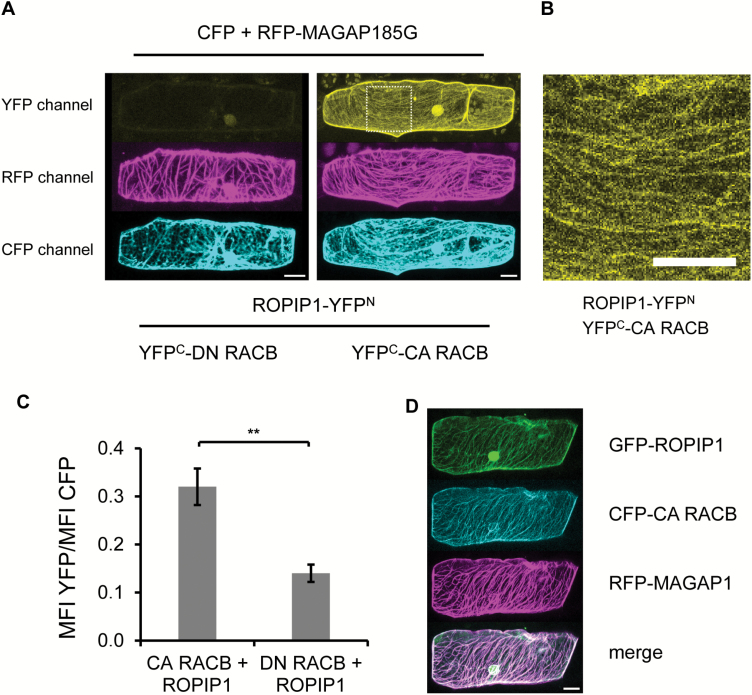
Split YFP complementation of ROPIP1–YFP^N^ and YFP^C^–CA HvRACB *in planta*. (A) ROPIP1–YFP^N^ was transiently co-expressed with DN or CA (right) YFP^C^–HvRACB, the inactive RFP–HvMAGAP1-R185G mutant, and CFP as a transformation marker in barley leaf epidermal cells. Confocal laser scanning microscopy whole-cell maximum projections are shown. (B) Detailed picture of the ROPIP1–YFP^N^ and YFP^C^–CA HvRACB co-expressing cell from (A) (dashed square). A maximum projection of 10 optical sections at 2 µm from the upper cell cortex is shown. Scale bars in (A) and (B) are 20 µm. (C) Ratiometric measurement of YFP fluorescence complementation. ROPIP1–YFP^N^ was transiently co-expressed with YFP^C^–CA HvRACB or YFP^C^– DN HvRACB, and YFP signals were normalized to signals from co-expressed CFP. Error bars are ±SE. Two-sided Student’s *t*-test (**P≤0.01). (D) Co-expression of GFP–ROPIP1, CFP–CA HvRACB, and RFP–HvMAGAP1. Transformed cells were imaged as whole-cell scans by confocal laser scanning microscopy at 48 hat. GFP–ROPIP1, CFP–CA HvRACB, and RFP–HvMAGAP1 showed similar localization at the cell periphery and at microtubules. The scale bar is 20 µm.

To support that ROPIP1 can interact with activated HvRACB *in planta*, we performed ratiometric BiFC (Fig. 6A–C) (Miller *et al.*, 2015). Therefore, we fused the N-terminal part of YFP (YFP^N^) to ROPIP and the C-terminal part (YFP^C^) to different versions of HvRACB. ROPIP1–YFP^N^ was transiently co-expressed with either YFP^C^–CA HvRACB or YFP^C^–DN HvRACB, RFP–HvMAGAP1-R185G, a mutant lacking the catalytic arginine finger of GAP domains (Hoefle *et al.*, 2011), and CFP. The RFP–HvMAGAP1 R185G mutant was chosen as its co-expression with ROPIP1 was seen to influence the organization of the cortical MT network less than co-expression of RFP–HvMAGAP1, which destabilized MTs in the presence of ROPIP1. However, RFP–HvMAGAP1 R185G interacts with CA HvRACB *in planta* (Hoefle *et al.*, 2011), and GFP–ROPIP1 was recruited to MTs by RFP–HvMAGAP1-R185G (Supplementary Fig. S8). Ratiometric measurement of YFP versus CFP signals showed fluorescence complementation of ROPIP1–YFP^N^ with YFP^C^–CA HvRACB but only weakly with YFP^C^–DN HvRACB or YFP^C^–HvMAGAP1 (Fig. 6A: Supplementary Fig. S9). The mean YFP/CFP ratio of YFP^C^–CA HvRACB-co-expressing cells was significantly different from that in YFP^C^–DN HvRACB-co-expressing or YFP^C^–HvMAGAP1 cells (*P*≤0.01 or 0.001, respectively Student’s *t*-test; Fig. 6C; Supplementary Fig. S9). The BiFC signal of ROPIP1–YFP^N^ and YFP^C^–CA HvRACB was predominantly observed at the cell periphery and as filamentous strings at the cell cortex, probably representing cortical MTs (Fig. 6B, D). Localization at the cell periphery is indicative for the plasma membrane, as activated HvRACB is partially plasma membrane associated (Schultheiss *et al.*, 2003). This supported a direct protein–protein interaction of ROPIP1–YFP^N^ and YFP^C^–CA HvRACB but not with YFP^C^–DN HvRACB or YFP^C^–HvMAGAP1 *in planta*. Localization of the BiFC signal at filamentous structures suggested that ROPIP1, activated HvRACB, and HvMAGAP1 are simultaneously present at MTs, when co-expressed. This was supported by co-localization of GFP–ROPIP1, CFP–CA HvRACB, and RFP–HvMAGAP1 at both MTs and the cell periphery (Fig. 6D).

### ROPIP1 causes microtubule network destabilization

**Fig. 7.**
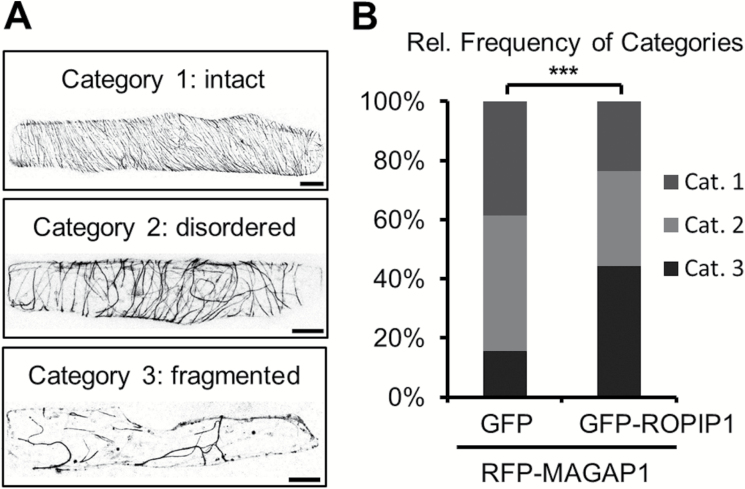
Co-expression of GFP–ROPIP1 and RFP–HvMAGAP1 enhanced microtubule (MT) network disorganization. (A) Example micrographs illustrating three distinct categories of MT network organization in barley epidermal cells. Confocal laser scanning microscopy whole-cell projections of barley epidermal cells transiently co-expressing GFP–ROPIP1 and RFP–HvMAGAP1 are shown in gray scale. Scale bars are 20 µm. (B) Mean relative frequencies of the categories at 12–24 hat. The absolute numbers of cells per category of *n*=145 GFP and *n*=132 GFP–ROPIP1 cells each co-transformed with RFP–HvMAGAP1 obtained from four independent repetitions were compared in a χ^2^ test (***P≤0.001; χ^2^=27.92; df=2). Cells of category 3 exhibiting a heavily disorganized MT network tripled from 15.5% in the GFP control to 44.3% in cells expressing GFP–ROPIP1.

MTs reorganize towards the site of attempted entry by *Bgh* (Hoefle *et al.*, 2011). We hence asked whether the recruitment of ROPIPI1 to MTs by HvMAGAP1 could influence MT organization. RFP–HvMAGAP1 was co-bombarded into barley epidermal cells with either GFP–ROPIP1 or GFP as control. We scored MT organization in three categories: intact MT network, disordered MT network, or fragmented MT network (Fig. 7A). Co-expression of GFP–ROPIP1 together with RFP–HvMAGAP1 led to a highly significant change (*P*≤0.001, χ^2^ test) in the distribution of the three categories when compared with control cells (Fig. 7B). The relative amount of category 3 cells exhibiting a fragmented MT network tripled from 15% in control cells to ~45% in cells co-expressing GFP–ROPIP1 and RFP–HvMAGAP1.

## Discussion

We identified the retroelement-encoded peptide ROPIP1 of *Bgh* that shows the potential to interact with the barley S-factor HvRACB and to promote fungal penetration success on barley. Some *B. graminis* effectors have recently been characterized. Direct interaction with potential host target proteins has been reported for CSEP0055 that interacts with the barley pathogenesis-related protein PR17c (Zhang *et al.*, 2012) and for CSEP0105 and CSEP0162 that interact with the small heat shock proteins 16.9 and 17.5 (Ahmed *et al.*, 2015). *Bgh* BEC3 and BEC4 were shown to interact with the host proteins thiopurine methyltransferase, an ubiquitin-conjugating enzyme, and an ADP ribosylation factor-GTPase-activating protein (Schmidt *et al.* 2014). Additionally, in a combination of protein pull-down and Y2H experiments, CSEP0064 interacted with a glutathione *S*-transferase, a malate dehydrogenase, and a pathogenesis-related-5 protein isoform (Pennington *et al.*, 2016). Some *B. graminis* effector candidates do not possess N-terminal signal peptides for secretion, though they are thought to act intracellularly. This is the case for the class of EKA effectors (Ridout *et al.*, 2006) and candidate effector proteins (CEPs) of the wheat powdery mildew *Bgt* (Wicker *et al.*, 2013). EKA effector genes are evolutionarily and transcriptionally linked with autonomous non-LTR retroelements (Ridout *et al.*, 2006; Sacristán *et al.*, 2009), whereas CSEP genes are surrounded by non-autonomous non-LTRs such as *Eg-R1* and *Egh24* (Pedersen *et al.*, 2012). Recent findings suggest that EKAs evolved from insertions of premature stop codons in LINE ORF1 protein (ORF1p), which subsequently underwent positive selection (Amselem *et al.*, 2015). This further supports potential neo-functionalization of *Bgh* retroelements as a genetic resource for the evolution of novel effector proteins.

The ROPIP1 sequence is distributed in the genome of *Bgh* by Eg-R1 but does not encode an N-terminal signal peptide. The N-terminal ROPIP1 sequence part is not equipped with a canonical start codon on Eg-R1, whereas ROPIP1-Cter could be translated from an internal ATG. This raises the future question of whether there might be a gain of function through formation of chimeric ORFs or whether the C-terminal peptide ORF ROPIP1-Cter represents the actual effector. Inspection of the *Bgh* genome readily revealed the presence of several chimeric ORFs which encoded extended stretches of amino acids and partially N-terminal signal peptides that are in-frame with ROPIP1 (Supplementary Table S1). Due to the repetitive nature of ROPIP1 and the consequent presence of thousands of copies, the genomic origin of the detected polyadenylated RNAs and the corresponding protein remain unresolved and need further investigations. Our western blot experiment suggested that a ROPIP1-related sequence indeed is translated into protein, because the antibody used against a ROPIP1-Cter peptide detected both recombinant ROPIP1 and a single protein which was only present in *Bgh*-infected leaves. The apparent shift in mobility of *E. coli*-expressed recROPIP1 and the native ROPIP1 signal might be explained in different ways. Possibly, a single 5'-extended chimeric ORF is translated in *Bgh* and detected here. The higher molecular weight could also be explained by post-translational modification of ROPIP1. Alternatively, Eg-R1 transcripts translate as ROPIP1-Cter only and form SDS-stable oligomers. Indeed, the HHpred server (Söding *et al.*, 2005) for protein remote homology detection and 3D structure prediction detects that ROPIP-Cter shows weak similarity to functionally diverse YigF proteins from pro- and eukaryotes that have the ability to form homodimers or homotrimers (Deriu *et al.*, 2003). Immunogold labeling and TEM further supported that this protein is secreted by the fungus and translocated into the host cell. Protein signal appeared in the infecting fungus and infected cells, but did not appear in either uninfected barley or the mesophyll of infected barley. Hence, a host-translocated and intracellularly acting protein of *Bgh* was detected by the α-ROPIP1 antibody. Since α-ROPIP1 also detected recombinant ROPIP1 expressed from *E. coli*, we suggest that ROPIP1 or a ROPIP1-related protein was detected in the fungus and the host cell cytoplasm. Further, ROPIP1 interacted with the barley S-factor HvRACB in yeast and *in planta*. Hence, the barley small GTPase HvRACB is probably the host target of a ROPIP1 effector. Some first insights into a possible mode of action of ROPIP1 were gained. GFP–ROPIP1 co-located with CFP–HvRACB and HvRACB-interacting RFP–HvMAGAP1 at cortical MTs in barley epidermal cells. Transient overexpression of GFP–ROPIP1 together with RFP–HvMAGAP1 promoted the breakdown of the cortical MT array. Although this dramatic effect may be attributed to overexpression of ROPIP1, we hypothesize that release of ROPIP1 from appressoria creates a sufficient concentration for spatially restricted effects on MTs. MTs are involved in penetration resistance to powdery mildew fungi, but MT structure is locally diffuse, where *Bgh* penetrates. Additionally, HvRACB and HvRACB-like ROP GTPases are key regulators of MTs (Kobayashi *et al.*, 1997; Hoefle *et al.*, 2011; Huesmann *et al.*, 2012). The potential manipulation of host MT arrays by ROPIP1 could either inhibit polarized cell wall-associated defense or facilitate fungal invasion and membrane delivery for formation of the extrahaustorial membrane and matrix (Dörmann *et al.*, 2014).

### ROPIP1: a *Bgh* effector of retroelement origin

ROPIP1 does not fit pre-defined categories or definitions of secreted effector proteins of filamentous plant pathogens, or prokaryotic or eukaryotic pathogens in general. However, there are recent published examples that expand the current model of plant pathogen effectors beyond strict definitions. The effectors PsIsc1 and VdIsc1 of the oomycete *Phytophthora sojae* and the phylogenetically distinct true fungus *Verticillium dahliae*, respectively, attenuate the PTI response by misdirecting the synthesis of the plant defense hormone salicylic acid. Neither protein encodes N-terminal signal peptides for secretion, and PsIsc1 can functionally replace the N-terminal signal peptide and the RXLR-dEER host translocation motif of the effector Avr1b of *P. sojae* (Liu *et al.*, 2014). This adds to the assumption that there should be an additional secretion pathway besides the conventional co-translational loading into the endomembrane route or a process of cytoplasm exchange with host cells in filamentous plant pathogens possibly involving exosome release from multivesicular bodies (Micali *et al.*, 2011).

ROPIP1 constitutes an unconventional effector candidate whose evolution was possibly supported by the high repeat content of the *Bgh* genome. Sequences similar to ROPIP1 can be found in *Bgt* but no clear ROPIP1/Eg-R1 homologs are present in sequenced genomes of powdery mildew fungi from dicots. It would be of great interest to learn whether there are further repeat-encoded proteins being expressed in other species. The finding of long intergenic non-coding (linc) RNAs being translated in the human proteome provoked the view that presumably non-coding RNAs constitute an evolutionary playground (Wilhelm *et al.*, 2014). Similarly, ribosome profiling identified 5' regions of ~10–100 codons of yeast long non-coding RNAs to be bound by ribosomes, which suggests their translation (Smith *et al.*, 2014). By looking at ROPIP1, we are possibly observing the neo-functionalization of a non-coding retroelement into a new effector gene. The nature of the Eg-R1 element has to be characterized further as it shares some properties of SINEs but, different from SINEs, it is obviously transcribed by RNA polymerase II (Wei *et al.*, 1996; this study).

The *Bgh* genome is largely composed of TEs, with genes being interspersed in small clusters. It is one of the biggest ascomycete genomes possibly due to the absence of a TE spread controlling the repeat-induced point mutation (RIP) mechanism (Spanu *et al.*, 2010). The high repeat content may give myriads of options for non-allelic recombination, making the genome very dynamic. The current knowledge is too sparse to draw a clear conclusion on the evolution of a possibly virulence-promoting sequence being dispersed throughout the genome by a SINE-like retroelement. In any case, the experimental data suggest an effector function of a ROPIP1 sequence-containing protein. It further appears possible that ROPIP1 gained an N-terminal signal peptide by insertional formation of chimeric ORFs like those exemplarily identified in this study (Supplementary Table S1). Even if this should not be the case, ROPIP1 or ROPIP1-Cter yielded scores for predicted non-classical protein secretion comparable with those of PsIsc1 and VdIsc1 using the SecretomeP 2.0 server (Bendtsen *et al.*, 2004) in analogy to Liu *et al.* (2014). Predicted protein folding (Supplementary Fig. S4) but absence of predictable functional domains in ROPIP1 is typical as many effector proteins represent novel folds which implies the possibility that they are not derived from sequence variation of pre-existing genes. Further, gene losses of the primary and secondary metabolism of *B. graminis*, probably due to high retrotransposon activity, reflect its extreme obligate biotrophic lifestyle (Spanu *et al.*, 2010; Wicker *et al.*, 2013) which is likely to enhance selective pressure. In a genome with a reduced gene set, non-gene transcripts may gain novel functionalities in virulence and in general.

## Supplementary data

Supplementary data are available at JXB online.

Table S1. Genomic ROPIP1 sequence variants with signal peptide prediction.

Table S2. Nucleotide and amino acid sequences of ROPIP1 and Eg-R1.

Table S3. List of oligonucleotides used in this study

Fig. S1. Sequence alignments of Eg-R1, ROPIP1, and ROPIP1-Cter.

Fig. S2. Exemplary genomic insertion and hypothetical architecture of the Eg-R1 retroelement.

Fig. S3. Semi-quantitative reverse transcription–PCR of *ROPIP1.*

Fig. S4. Genomic insertion size distribution of Eg-R1, 5' end similarity of BG_non-LTR elements, and secondary and tertiary structure predicton of ROPIP1.

Fig. S5. Targeted assays showing preferential and specific protein interaction of Bgh ROPIP1 with wild-type (WT) RACB and CA RACB.

Fig. S6. Test of silencing capacity of ROPIP1-RNAi and sequence alignment of ROPIP1 and ROPIP1-RNAi-resuce.

Fig. S7. Immunogold labeling of α-ROPIP1 in mesophyll cells of *Bgh*-infected barley leaves.

Fig. S8. R185G mutation of HvMAGAP1 does not alter microtubule association of GFP–ROPIP1.

Fig. S9. HvMAGAP1 does not interact with ROPIP1 in a split YFP complementation assay.

supplementary Tables S1-S3 and Figures S1-S9
