# Supplementary material for: A barley powdery mildew fungus non-autonomous retrotransposon encodes a peptide that supports penetration success on barley
Source: J Exp Bot. 2018 May 11;69(15):3745–58. doi: 10.1093/jxb/ery174 (PMC6022598; doi:10.1093/jxb/ery174)
Supplement: supplementary Tables S1-S3 and Figures S1-S9 [file ery174_suppl_supplementary-tables_figures.pdf]

Supplementary data to Nottensteiner et al. JXB

Table S1. Genomic ROPIP1 Sequence Variants with Signal Peptide Prediction.

Signal Peptides were predicted using the SignalP server in the indicated version [86,87]. EgR1extORF1 till EgR1extORF5 were obtained from manually inspecting the topmost 45 BLASTn hits for 5'-elongated ROPIP1-ORFs using the ROPIP1 nucleotide sequence as query against the BGH DH14 Genome v3b database of *Bgh* genomic DNA contigs (blugen.org). EgR1extORF6 was found among the manually inspected 10 topmost BLASTn hits of the ROPIP1 query against the NCBI Trace archive database 'Blumeria graminis f sp hordei WGS' that contains *Bgh* whole genome shotgun raw reads. The 3'-truncated EgR1extORF7 was found in vicinity to an AVRa10-like effector protein by using the ROPIP1 nucleotide query against the NCBI non-redundant nucleotide collection database. EgR1extORF8 contains a putative intron between the predicted signal peptide and the ROPIP1 sequence. Sequence extensions of ROPIP1 contributing to a positive signal peptide prediction are underlined. EgR1extORF2, EgR1extORF3, EgR1extORF5 and EgR1extORF8 were amplifiable from genomic DNA prepared from *Bgh* race A6-infected barley leaves using gene-specific primers.

#	Source	Identifier	Length	SignalP	Name
1	BluGen	CAUH01000529:1..1000	106	v3.0	EgR1extORF1
2	BluGen	CAUH01001259:1..1000	91	v3.0	EgR1extORF2
3	BluGen	CAUH01002559:9000..11000	88	v3.0	EgR1extORF3
4	BluGen	CAUH01002841:27000..29500	105	v3.0 & v4.1	EgR1extORF4
5	BluGen	CAUH01010509:1..1419	108	v3.0	EgR1extORF5
6	NCBI Trace Archive	ti|2268612253	118	v3.0	EgR1extORF6
7	NCBI Nucleotide Collection	GenBank: EU098096.1	74	v3.0 & v4.1	EgR1extORF7
8	BluGen	CAUH01002575:2300..3800	133	v3.0	EgR1extORF8

Name	Sequence
EgR1extORF 1	MNPPLILPGFSPFLVCGDFYCLSLPSFGGLFSTPVSGVPYEPPALTVESAEPRLSNNLLTSMRIPSRLRDLYRLHFSSHPPITIIMKLTTIRSDVRVEALLVTNP*
EgR1extORF 2	MDCRLEGVVLGILGGLFSTPVSGVPYEPPALTVESAEPRLSNNLLTSMRIPSRLRDLYRLHFSSHPPITIIMKLTTIRSDVRVEALLVTNP*
EgR1extORF 3	MWWNFAPVAGGLFSTPVSGVPYEPPALTVESAEPRLSNNLLTSMSIPSRLRDLYRLHFSSHPPITIIMKLTTIRSDVRVEALLVTNP*
EgR1extORF 4	MAGDQWGKLLFAIGVTSLLVAEARWSGGLFSTPVSGVPYEPPALTVESAEPRLSNNLLTSMRIPSRLRDLYRLHFSSHPPITIIMKLTTIRSDVRVEALLVTNP*
EgR1extORF 5	MTGTLFTYKMTLLTLETLLLWISIANTTMGGGLFSTPVSGVPYEPPALTVESAEPRLSNNLLTSMRIPSRLRDLYRLHFSSHPPITIIMKLTTIRSDVRVEALLVTNP*
EgR1extORF 6	MFFSSLAPQAHYSWLAPLAPKLWQTWSGKRPFHTLHPPLGGGLFSTPVSGVPYEPPALTVESAEPRLSNNLLTSMRIPSRLRDLYRLHFSSHPPITIIMKLTTIRSDVRVEALLVTNP*
EgR1extORF 7	MGRSSGGHPALVAACSCDFLLGAAGGLFSTPVSGVPYEPPALTVESAEPRLNDKVLTSMRIPKRFRGLYRLHP*
EgR1extORF 8	MNHLHGLQSQSKIWIISWMDPFFLVRGKPVLSVDLSTQHTMVLGSNEFQMKQVTSGLFSTPVSGVPYEPPALTVESAEPRLSNNLLTSMRIPSRLRDLYRLHFSSHPPITIIMKLTTIRSDVRVEALLVTNP*

**Table S2: Nucleotide and Amino Acid Sequences of ROPIP1 and Eg-R1.**
**Nucleotide and amino acid sequences of ROPIP1 and related sequences**
**Nucleotide sequence of ROPIP1. Please note, the underlined ATG is artificial. The ATG in red corresponds to the in frame start codon of ROPIP1-Cter, the TGA in blue represents the STOP codon.**<br><br>>ROPIP1<br><br>**ATG**TCAACACCTGTCAGTGGTGTCCCCTACGAACCTCCAGCTCTCACTGTAGAGTCTGCAGAGCCAAGATTGAGTAACAACCTCCTCACTTCG**ATG**AGGATTCCCAGTCGCCTTCGCGATCTGTATCGTCTTCATTTCTCATCACATCCCCCTATCACCATTATCATGAAGCTAACCACGATCAGATCAGACGTTAGGGTCGAAGCCCTTCTCGTCACCAACCCT**TGA**
**Amino acid sequence of ROPIP1. Please note, the underlined M is artificial. The M in red corresponds to first methionine in frame and represents the start of ROPIP1-Cter**<br><br>>ROPIP1-translated<br><br>MSTPVSGVPYEPPALTVESAEPRLSNNLLTS**M**RIPSRLRDLYRLHFSSHPPITIIMKLTTIRSDVRVEALLVTNP-
**Nucleotide sequence of ROPIP1-Cter.**<br><br>>ROPIP1-Cter<br><br>**ATG**AGGATTCCCAGTCGCCTTCGCGATCTGTATCGTCTTCATTTCTCATCACATCCCCCTATCACCATTATCATGAAGCTAACCACGATCAGATCAGACGTTAGGGTCGAAGCCCTTCTCGTCACCAACCCT**TGA**
**Amino acid sequence of ROPIP1-Cter.**<br><br>>ROPIP1-Cter-translated<br><br>**M**RIPSRLRDLYRLHFSSHPPITIIMKLTTIRSDVRVEALLVTNP-

Annotated nucleotide sequence of Eg-R1. GenBank: X86077.1.

>gi|763091|emb|X86077.1| E.graminis mRNA for a retroposon-type repetitive element

CTCAACACCTGTCAGTGGTGTCCCCTACGAACCTCCAGCTCTCACTGTAGAGTCTGCAGAGCCAAGATTGAGT
AACAACCTCCTCACTTCG**ATG**AGGATTCCCAGTCGCCTTCGCGATCTGTATCGTCTTCATTTCTCATCACATC
CCCCTATCACCATTATCATGAAGCTAACCACGATCAGATCAGACGTTAGGGTCGAAGCCCTTCTCGTCACCAA
CCCT**TGA**TCATGGAGGAAAAGCCATCCGATGAGTTTCCGGAGCGAACCCAGCACCCGATACAGAGATGGTGGA
TTGGATCAAGATGGCCTCGAATGCTACAAAGAAGGGAGATATAGATGAATATGCGCACCTCATGTGGTCCCGG
TGTCTAGGCCTCGCCTGGCTGGGGCTGCAGGTTTTTTGAGACTTTTTCCCGCGCACGCAGTTTCCATTGCTAC
CATTTTTATTTTTTTTTGGGTAGAAGGTTCCGTGGAAAAGGTGGCTGAATTCCACGGGTAAATACTGAGCTGA
ATGGCTATTCATCATGGGCAATATATCAATGATCTTACAAGTAACAAGAGAGGACAGGAGCACTGTACAAGGT
GTGGCCTGGGCCAGAGAGGAGCCCAATGTGCTAGATAGTCGAAGACTCAAGTAGTACAATAAAAACCCACCCA
CTCATTACTCCAAAAAAAAAAAAAAAAAAA

Consensus sequence of 23 manually inspected full-length genomic insertions of Eg-R1 identified by target site duplications in the Genome v3b (contigs) Database of BluGen. The 11 underlined nucleotides are absent in the Eg-R1 annotation (X86077.1).

>Eg-R1_CONSENUS

-----
gGGGGACTATTCTCAACACCTGTCAGTGGTGTCCCCTACGAACCTCCAGCtCTCaCTgtAgAGTCTGCAGAGC
CaAGATTGAGTaACAAccTCCTCACTtCG**ATG**AGGaTTCCCAgTCGCcTTCGCgaTCTGTATCgTCTTCATtt
cTcAtCACATCcCCcTATCACCATTATCATGAAGCTAACCACgATCAGATCAGAcGtTAgggTCgaAGCCCTt
CTcgTCACCAACCCT**TGA**TCATGGAgGAAAAGCCATCCgATGAGTTTCCGGAGcGaACCCAGCACCCGATACg
gAGATGGTGGATTGGATCAAGatGGCcTCGAaTgcTAcAAAGAAGGGAGATATAGATGAATATGCGCACCTCa
TGTGGTCCCGGTGTCTAGGCCTCGCCTGGCTGGGGCTGCAGGTTTTTTGAGACTTTTtCCCgCGCACgcAGTT
TCCATTGCTACCattttattttTTT-GGGTAGAAGGTTCCgTGGAaAAGGTGgCTGAATTCcA-
GGGTAAATACTGAGCTGAATgGCTAtTCATCA-
gGGCAAtaTAtCAATGATCTTaCAAGtAACAagAGAGGacAgGAGCACTGTACAAGgTGtGGccTGGGCCAGA
GAGGAgCCCAATGTGCTAgATAGTCgaaGACTCAAgTAGTACAATAAA--AaCcCACtcactcac--------
------

An exemplary 5'-RACE PCR-derived sequence using primers annealing to the ROPIP1 part of Eg-R1. The crossed-out Oligo (T) stretch derived from process-dependent A-tailing. Underlined nucleotides are identical to the first 11 bp of Eg-R1 CONSENSUS.

>5-RACE_cand_2-1

~~TTTTTTTTTTTTTTTT~~GGGGGACTATTCTCAACACCTGTCAGTGGTGTCCCCTACGAACCTCCAGCTCTCACT
GTAGAGTCTGCAGAGCCAAGATTGAGTAACAACCTCCTCACTTCG**ATG**AGGATTCCCAGTCGCCTTCGCGGTC
TGTATCGTCTTCATTTCTCATCACATCCCCCTATCACCATTATCATGAAGCTAACCACGATCAGATCAGAC

Table S3. List of Oligonucleotides Used in this Study.	
**Name**	**Sequence 5‘→3‘**
V42fwd	ACCTGTCAGTGGTGTCCC
V42rev	CAAGGGTTGGTGACGAGAAGG
B8B,V21B_BamH1fwd	GGGGATCCATGTCAACACCTGTTAGTGGT
V20A,V42ABamH1fwd	GGGGATCCATGTCAACACCTG
V42A_SmaI_F	CCCGGGATGTCAACACCTGTCAGTGGTG
V42A,V20Bsalrev	TCAGTCGACCGGGTTGGTGACGAG
V42A,V20Brev	CATGATCACGGGTTGGTGAC
F-V42ACter_Sma	CCCGGGATGAGGATTCCCAGTC
R_V42ACter_Bam	GGATCCTCAAGGGTTGGTGACGAG
V42A,V20B-BamH1kurz	AACCTCCTCGGATCCATGAGGATTCC
R_V42A_Nter_BamHI	GGATCCTCACGAAGTGAGGAGGTTGTTAC
V42A-SP2	GTCTGATCTGATCGTGGTTAGC
V42A-SP3	AGACGATACAGATCGCGAAGGC
S42A_EcoRI-fwd-1	GAATTCGGGCTAATGAATCCGCCT
S42A_EcoRI-fwd-2	GAATTCGTGGATGGATTGTCGGCT
S42A_EcoRI-fwd-3	GAATTCTATGTGGTGGAATTTCGC
S42A_EcoRI-fwd-4	GAATTCATGGCCGGTGATCAATGG
S42A_EcoRI-fwd-5	GAATTCGCTATGACACATGACCGG
TW42A_F	ATGAATCATCTTCATGGGTTGC
TW42A_R	TCAAGGGTTGGTGACGAG
TW42A_Intron_F2	CAGATGAAACAAGTAACTAGGG
HvUBC2_fwd	TCTCGTCCCTGAGATTGCCCACAT
HvUBC2_rev	TTTCTCGGGACAGCAACACAATCTTCT

T-PR1b/3´-2	AGGTGTTGGAGCCGTAGTC
T-PR1b/5´-2	AAGCTGCAAGCGTTCGCC
*Bgh*_beta-tub_F	TCTGCCATTTTCCGCGGTAA
*Bgh*_beta-tub_R	CGTTGCTTACTTCCTCTGGA

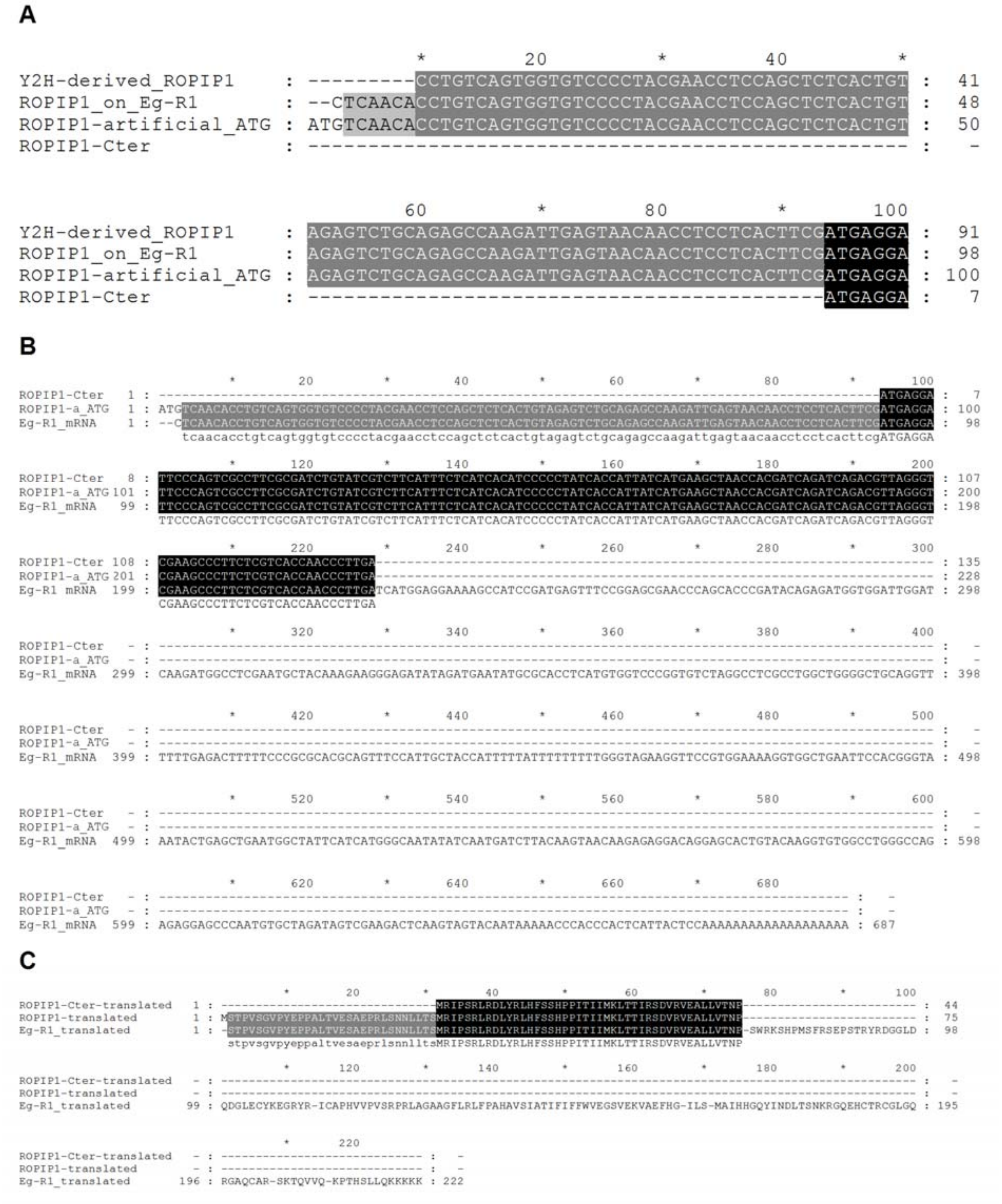

Fig. S1. Sequence Alignments of Eg-R1, ROPIP1 and ROPIP1-Cter.

(A) Nucleotide sequence alignment of the 5'-ends of the Y2H-derived ROPIP1 sequence, the annotated EgR1 (X86077.1) sequence, the ROPIP1 sequence equipped with an artificial 5'-ATG and the start of the intrinsic ORF represented by ROPIP1-Cter. (B) Nucleotide sequence alignment of Eg-R1, ROPIP1-a_ATG (equipped with an artificial 5'-ATG) and ROPIP1-Cter. (C) Amino acid sequence alignment of Eg-R1, ROPIP1 and ROPIP1-Cter.

>CAUH01002330:34805..35675

~~GCAGAAGATGTATCCAAAATGTGCGTGGTTATGGGGTTTTCCATGGGCTT~~

TSD

~~GAAACAGGCAGCTTCTGCTTGTTTTCGCAGGCGAGCG~~TCTGCGTTGATAT

New 5'-end ROPIP1 Eg-R1 (X86077.1)

GGGGGACTATTCTCAACACCTGTCAGTGGTGTCCCCTACGAACCTCCAGC

TCTCACTGTAGAGTCTGCAGAGCCAAGATTGAGTAACAACCTCCTCACTT

CGATGAGGATTCCCAGTCGCCTTCGCGATCTGTATCGTCTTCATTTCTCA

TCACATCCCCCTATCACCATTATCATGAAGCTAACCACGATCAGATCAGA

CGTTAGGGTCGAAGCCCTTCTCGTCACCAACCCTTGATCATGGAGGAAAA

A-Box

GCCATCCGATGAGTTTCCGGAGCGAACCCAGCACCCGATACGGAGA**TGGT**

B-Box t-RNA related region TRGY

GG**A**TTG**GATCAA**GATGGCCTCGAATGCTACAAAGAAGGGAGATATAGATG

NNANNNGWTCRANNC V-domain

AATATGCGCACCTCATGTGGTCCCGGTGTCTAGGCCTCGCCTGGCTGGGG

Oligo(T) Oligo(T)

CTGCAGG**TTTTTT**GAGAC**TTTTT**CCCGCGCACGCAGTTTCCATTGCTACC

Oligo(T) Oligo(T)

A**TTTTT**A**TTTTTTTT**-GGGTAGAAGGTTCCGTGGAAAAGGTGGCTGAATTC

CATGGGTAAATACTGAGCTGAATGGCTATTCATCAAGGGCAATATATCAA

TGATCTTACAAGTAACAAGAGAGGACAGGAGCACTGTACAAGGTGTGGCC

TGGGCCAGAGAGGAGCCCAATGTGCTAGATAGTCGAAGACTCAAGTAGTA

Poly(A) signal TSD

C**AATAAA**AACCCACTCACTCACTCACTCTGCGTTGAT~~CTGTCGGATAGCA~~

~~CTTACTTGGTCCTTGATCTTTGGGGGGCCGGAGGGCTTGGGTCGGAGGTC~~

~~GCAGCTGTCATTGTCCGCGGA~~

Fig. S2. Exemplary Genomic Insertion and Hypothetical Architecture of the Eg-R1 Retroelement.

This exemplary genomic insertion of a full-length Eg-R1 retroposon was extracted from contig CAUH010023330 at nucleotide position 34805-35675 of the *Bgh* genome (blugen.org). The surrounding genomic sequence is stroked-through. Likely retrotransposition-derived target site duplications (TSDs) are highlighted in orange. The 5'-end extension of 11 bp missing in the Eg-R1 annotation (X86077.1) is highlighted in green. Nucleotides identical to Eg-R1 are underlined. Nucleotides identical to ROPIP1 are shaded in grey. A putative SINE-like region following the ROPIP1 sequence part was identified by following the protocol for SINE analysis of SINEBase (Vassetzky and Kramerov, 2012). A putative tRNA-Gln (tdbD00008587|Homo_sapiens|9606|Gln|CTG; sines.eimb.ru) -

related sequence region is highlighted in violet. Putative A- and B-Box-like sequences are shaded in yellow and turquoise. A-Box and B-Box pol III promoter consensus sequences determined for *Saccharomycetes* of the *Ascomycota* phylum (Marck et al., 2006) are given below the respective nucleotides. Matching nucleotides are in bold-type. Note that the putative A- and B-Box-like sequences are overlapping at one nucleotide highlighted in green. A region similar to the vertebrate SINE body part V-domain is highlighted in blue. Oligo(T)-stretches that would terminate pol III transcription are in bold-type. A likely functional polyadenylation (Poly(A)) signal is indicated in bold-type near the 3'-end of Eg-R1.

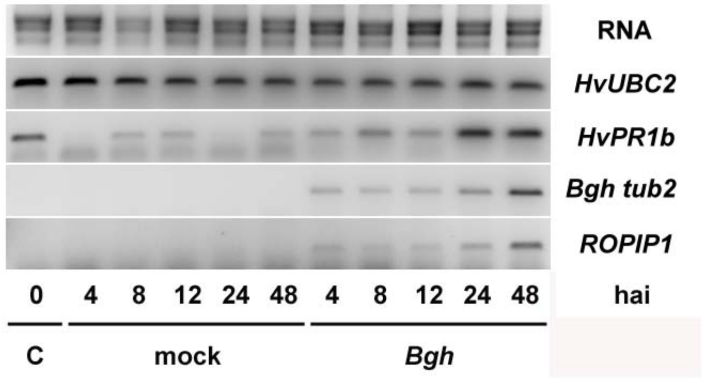

Fig. S3. Semi-Quantitative Reverse Transcription PCR of *ROPIP1*.

7d old barley primary leaves were inoculated with *Bgh* or mock treated. Leaves were cut and frozen in liquid N_2 at the indicated time points. Total RNA was extracted, DNAse I-digested and reverse transcribed into cDNA. Barley *Ubiquitin Conjugating Enzyme 2* (*HvUBC2*, AY220735.1) was amplified as control for cDNA quantity. Successful inoculation was checked by induction of the barley *Basic PR-1-Type Pathogenesis Related Protein* (*HvPR1b*, X74940.1) gene. *Blumeria graminis* f.sp. *hordei Tub2 Gene For Beta Tubulin* (*Bgh tub2*, AJ313149) was amplified to monitor the development of fungal biomass. The *ROPIP1* transcript appeared not to be induced after pathogen challenge. Being part of the SINE-like Eg-R1 retrotransposon the amplified ROPIP1 sequence is indistinguishable from Eg-R1. The experiment was repeated with similar results.

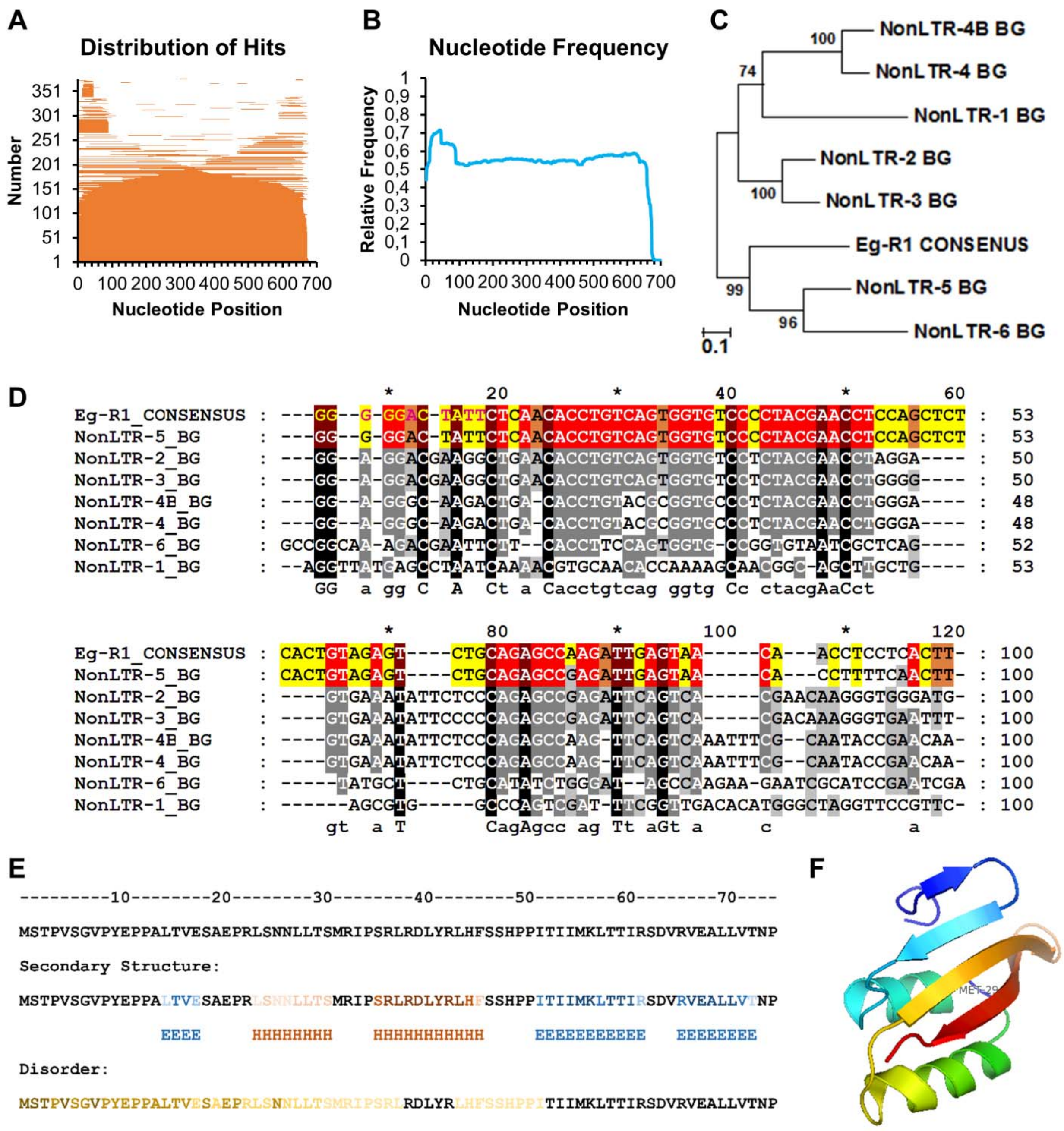

Fig. S4. Genomic Insertion Size Distribution of Eg-R1, 5'-End Similarity of BG_Non-LTR Elements, Secondary and Tertiary Structure Predicton of ROPIP1.

(A) Distribution of 376 BLASTn hits (BGH DH14 Genome v3b (contigs), blugen.org) using Eg-R1 (X86077.1) equipped with the 11 bp 5' sequence extension as query. The hits are shown sorted in size and plotted to their respective position on the query. (B) The frequency of nucleotides matching to the query was calculated. No preferential insertion of any Eg-R1 part was observed. The increased nucleotide frequency within the first 88 nucleotides is likely due to the high sequence similarity of this sequence part with NonLTR-5_BG (see D). Together with the pattern in A this might support unequal crossing over mediated by Eg-R1 as suggested by [10]. (C) A 670 bp Eg-R1 consensus sequence was generated by nucleotide sequence alignment of 23 Eg-R1 full-length genomic insertions being surrounded by manually searched for target site duplications. Genomic Eg-R1 CONSENSUS contained the 11 bp 5'-sequence extension but lacked the poly (A) tail of the annotated Eg-R1 mRNA

(X86077.1). 8 *Blumeria graminis* Non-LTR retrotransposons (BG_Non-LTRs) are deposited in Repbase Reports (2011, Volume11, Issue 9, [34]). EGRT1 Non-LTR Retrotransposon being identical to the annotated Eg-R1 sequence (X86077.1) was replaced by Eg-R1 CONSENSUS to calculate a phylogenetic tree by using the Maximum Likelihood method and 500 rounds of bootstrapping. (D) Nucleotide sequence alignment of the first 100 5'-nucleotides of the 8 BG_Non-LTRs. Eg-R1 CONSENSUS and NonLTR-5_BG shared a stretch of 88 identical nucleotides, except of one SNP, at their 5'-ends and were 93 % pairwise identical in the first 100 bp (identical residues are highlighted in color). All 8 BG_Non-LTRs were most identical (56 % pairwise identity) to each other within their first 100 bp. (E) ROPIP1 secondary structure prediction. Helix (H) and β-sheet (E) residues obtained from 4 algorithms provided by the Quick2D webserver [88] plus one obtained from the QUARK server [89] are shown combined. The darker the color the more algorithms predicted that residue. Results from protein disorder prediction using Quick2D [88] and DisEMBL [90] are depicted in a similar manner. Please note that the first Met of the ROPIP1 query is artificial. (F) *Ab initio* tertiary structure prediction of ROPIP1 obtained from the QUARK server [89]. The submitted query started with amino acid 5 (V) as depicted in E. Met 29 marks the start of ROPIP1-Cter in the model (amino acid position 32 in E). The estimated template modelling (TM-) score of the model was 0.3561 (TM-score ≥ 0.3: non-random structure).

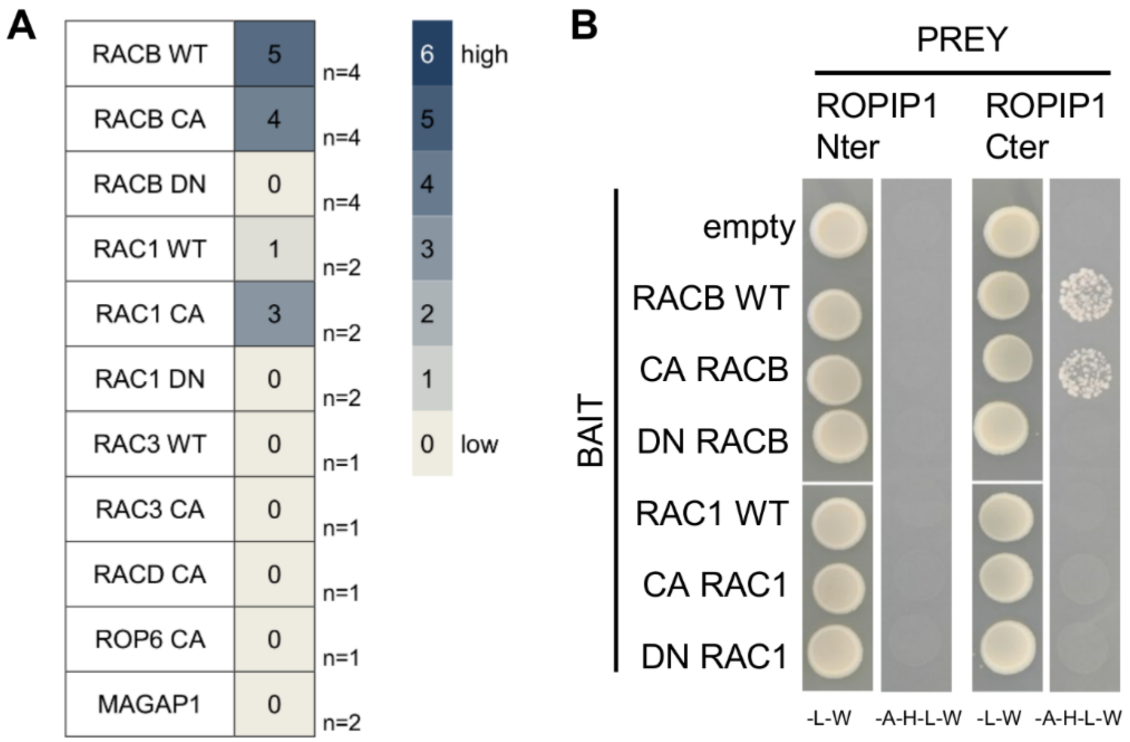

Fig. S5: Targeted Y2Hs showing preferential and specific protein interaction of Bgh ROPIP1 with RACB WT and CA RACB. (A) A protein interaction strength evaluation score based on growth of transformed yeast cells, expressing ROPIP1 as prey and baits as indicated, on media containing increasing HIS3 gene inhibitor concentrations (supplemented with 0, 0.5, 1.0, 1.5, 2.0, 2.5 mM 3-Amino-1,2,4-Triazole (3-AT)) was generated. A maximum score of 6 indicates strongest protein interaction, a minimum score of 0 no protein interaction in yeast. (B) Targeted Y2H with ROPIP1-Nter and ROPIP1-Cter as preys and bait vectors as indicated. The ROPIP1 sequence was split into an N-terminal (ROPIP1-Nter) and a C-terminal (ROPIP1-Cter) fragment. ROPIP1-Cter interacted with RACB (WT/CA) but not with RAC1. Left stripes: Transformation control medium (SD –L/-W); Right stripes: selectin medium (SD –A/-H/-L/-W). Drops of 10^6 cells per combination are shown.

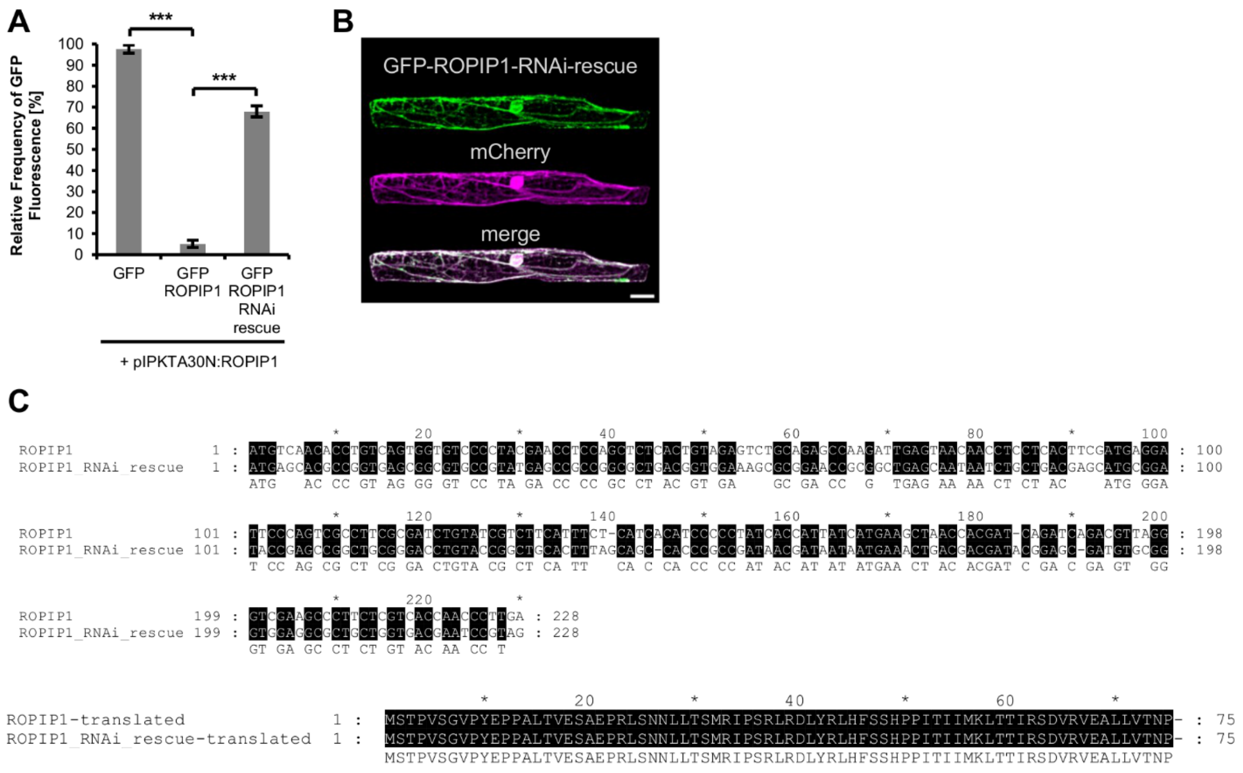

Fig. S6. Test of Silencing Capacity of ROPIP1-RNAi and Sequence Alignment of ROPIP1 and ROPIP1-RNAi-Resuce.

(A) Barley epidermal cells were transiently co-transformed with the RNAi vector pIPKTA30N-ROPIP1 (ROPIP1-RNAi) and pGY1-GFP or pGY1-GFP-ROPIP1 or pGY1-GFP-ROPIP1-RNAi-rescue plus pGY1-mCherry as transformation marker. To test the silencing capability 1.0 µg/shot pIPKTA30N-ROPIP1 together with 1.0 µg/shot pGY1-GFP, or 1.0 µg/shot pGY1-GFP-ROPIP1 (see below), or 1.0 µg/shot pGY1-GFP-ROPIP1-RNAi-rescue and 0.5 µg/shot pGY1-mCherry each were co-transformed into barley epidermal leaf cells. MCherry expressing cells were categorized into GFP fluorescence visible or not at 36 hat by fluorescence microscopy. The relative frequencies of GFP-fluorescence were compared in a two-sided Student's t-test. Transformed cells were identified by mCherry fluorescence and judged whether GFP fluorescence was visible or not at 36 hat by fluorescence microscopy. GFP-ROPIP1-RNAi-rescue was significantly less sensitive to ROPIP1-RNAi than GFP-ROPIP1. Bars are mean values of the relative frequencies of GFP to mCherry fluorescence from three independent experiments. In each experiment at least 150 cells per combination were scored. Error bars are ± SE. *** $P \leq 0.001$ (Student's t-test). (B) Confocal laser scanning microscopy whole cell projection of an exemplary barley epidermal cell showing translation of the GFP-ROPIP1-RNAi-rescue construct. The image was taken parallel to one repetition of (A). The co-transformed mCherry labeled the cytoplasm and the nucleoplasm. Co-localization is indicated by white pixels in the merge picture. Scale bar is 20 µm. (C) Nucleotide sequence and amino acid sequence alignment of the synthetic ROPIP1 RNAi rescue construct and ROPIP1. The codon usage of ROPIP1 was compared to the codon usage table of *Hordeum vulgare* subsp. *vulgare* in the codon usage database (kazusa.or.jp/codon/) using the graphical codon usage analyzer (gcua.schoedl.de/). Barley codons being most different, but not rare codons replaced the original codons of the ROPIP1 sequence. The

identity of the two sequences was reduced to 64%. The wobble base exchanges of the ROPIP1 RNAi rescue construct were silent.

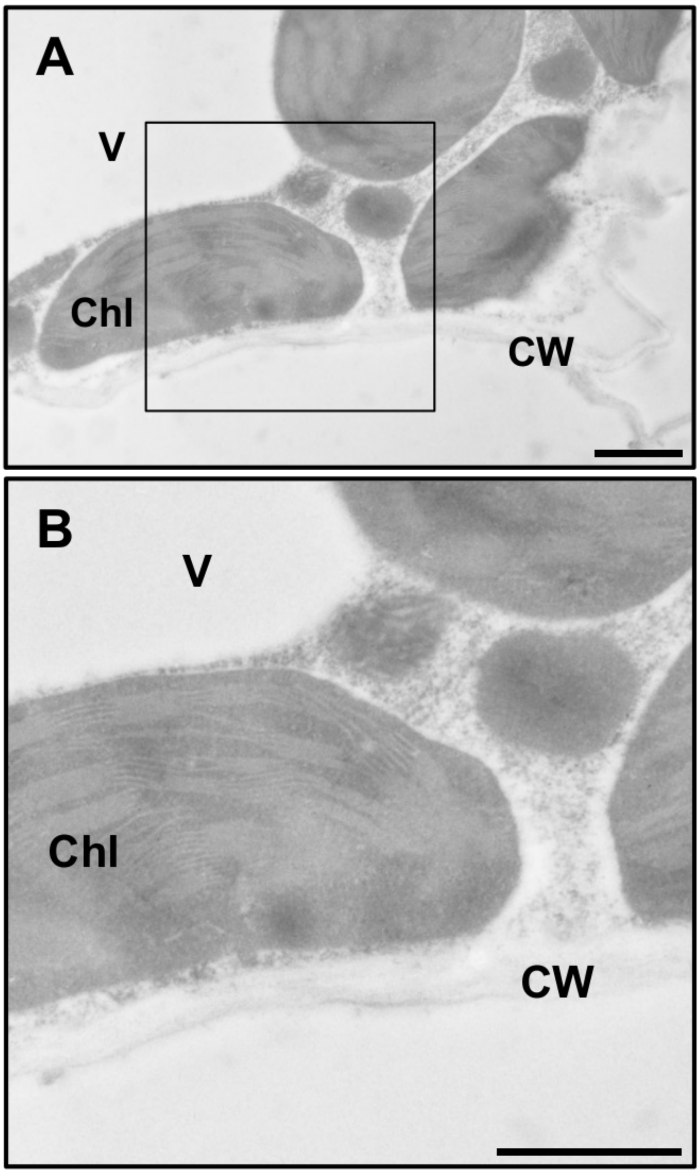

Fig. S7. Immunogold Labeling of α-ROPIP1 in Mesophyll Cells of *Bgh*-Infected Barley Leaves.
(A) Transmission electron micrograph of an ultrathin section of mesophyll cells of a *Bgh*-infected barley primary leaf at 3 dai. Gold-particles were almost absent throughout mesophyll cells after immunogold labeling of α-ROPIP1. (B) Detail picture of (A). Chl: chloroplast, CW: cell wall, V: vacuole. Scale bars are 1 μm.

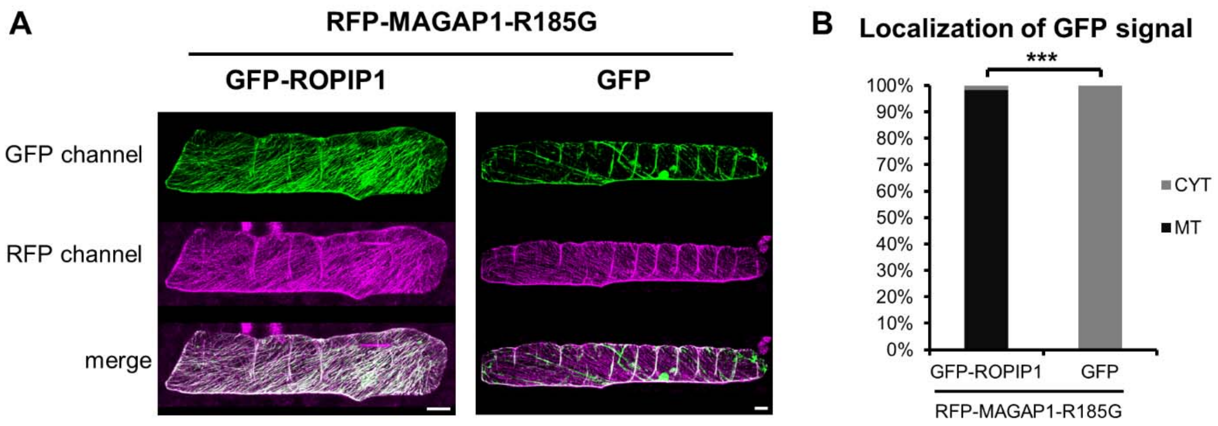

Fig. S8. R185G Mutation of HvMAGAP1 Does Not Alter Microtubule Association of GFP-ROPIP1.

Barley leaf epidermal cells were transiently transformed with RFP-HvMAGAP1-R185G plus GFP-ROPIP1 or GFP. HvMAGAP1-R185G lacks the catalytic arginine finger of its GAP domain. (A) Transformed cells were imaged as sequential whole cell scans by confocal laser scanning microscopy at 12 – 24 hat. (B) Maximum projections were categorized into GFP-fluorescence being located at microtubules (MT) or being absent from MTs but present in the cytoplasm (CYT). Bars represent relative frequencies of the categories derived from three independent replications. The respective absolute numbers per category of n = 59 GFP-ROPIP1 and n = 53 GFP expressing cells were compared in a X^2 test. *** $p \leq 0.001$ (X^2). Scale bars are 20 µm.

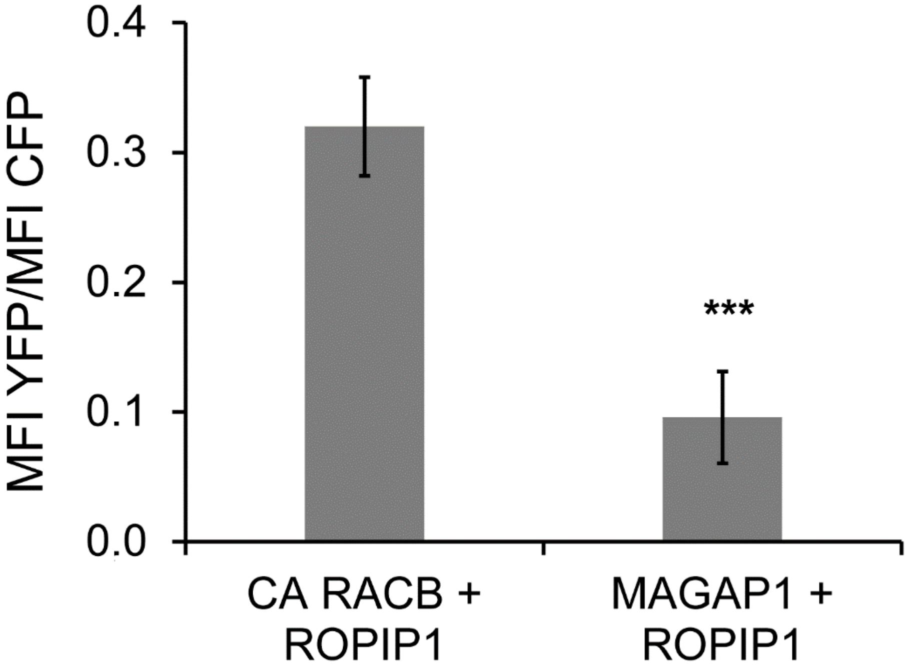

Fig. S9. HvMAGAP1 Does Not Interact with ROPIP1 in a Split YFP Complementation Assay. (A) ROPIP1-YFP^N was transiently co-expressed with CA YFP^C-HvRACB or YFP^C-HvMAGAP1 and CFP as transformation marker in barley leaf epidermal cells. Ratiometric measurement of YFP fluorescence complementation as normalized to signals from co-expressed CFP. Error bars are ± S.E.. Two-sided Student's t-test (***; $P \leq 0.001$).
